# Bipolar Fuzzy Pseudo-UP Ideal Of Pseudo-UP Algebra

**DOI:** 10.12688/f1000research.157173.1

**Published:** 2024-11-18

**Authors:** Alachew Amaneh Mechderso, Berhanu Assaye Alaba, Tilahun Mekonnen Munie, Teferi Getachew Alemayhu

**Affiliations:** 1 Department of Mathematics, Bahir Dar University College of Science, Bahir Dar, Amhara, 6000, Ethiopia; 2 Department of Mathematics, Debre Berhan University, Debre Birhan, Amhara, Ethiopia

**Keywords:** Pseudo-UP algebra, pseudo-UP ideal, Fuzzy pseudo-UP ideal, bipolar Fuzzy pseudo- UP ideal

## Abstract

In this paper, we apply the concept of bipolar fuzzy sets to pseudo-UP ideals in pseudo-UP algebras. We prove that the intersection of two bipolar fuzzy pseudo-UP ideals is also a bipolar fuzzy pseudo-UP ideal, while the union of two such ideals does not always result in a bipolar fuzzy pseudo-UP ideal. Additionally, we discuss the concepts of bipolar fuzzy pseudo-UP ideals under homomorphism and explore several related properties. The homomorphic image and inverse image of bipolar fuzzy pseudo-UP ideals in a pseudo-UP algebra are also examined in detail. Furthermore, we study the notion of a bipolar fuzzy pseudo-UP ideal under the Cartesian product of pseudo-UP algebra. The Cartesian product of any two bipolar fuzzy pseudo-UP ideals is also the bipolar fuzzy pseudo-UP ideal of pseudo-UP algebra, and then some related results are obtained. MSC: 03G25, 06D30.

## Introduction

Iseki ^1^ introduced BCK algebras. It is known that the class of BCK algebras is a proper subclass of the class of BCI-algebras. In 2020, Romano ^2^ introduced the concept of pseudo-UP ideals and pseudo-UP filters and derived basic properties. In 1965, Zadeh ^3^ popularized the concept of fuzzy sets. Since then, the concepts of fuzzy sets have been extensively used in many branches of mathematics. The fuzzifications of algebraic structures were initiated by Rosenfeld, ^4^ and he introduced the notion of fuzzy subgroups. In 2023, Mechderso ^5^ investigated the concept of fuzzy pseudo-UP ideals of pseudo-UP algebra. Wechler ^6^ studied how fuzzy algebraic structures plaid vital roles in mathematics with wide applications in many other branches such as computer sciences, theoretical physics, information sciences, control engineering, topological spaces and coding theory. For a fuzzy set, its degree of membership expresses its degree of containment of elements in it. Sometimes, the degree of membership also means the degree of satisfaction of elements to some property or constraint corresponding to a fuzzy set. ^7^ Keeping in view this notion, the membership degree 0 is in general assigned to those elements of the set that do not satisfy some property. In the usual study of fuzzy set representation, the elements with membership degree 0 are usually regarded as having the same characteristic.

Keeping in view these facts, Lee ^8^ proposed an extension of fuzzy sets named bipolar fuzzy sets. Lee ^9^ used the notion of bipolar fuzzy set and worked on bipolar valued fuzzy subalgebras and bipolar fuzzy ideals of BCK/BCI algebra. In recent times, Alaba ^10^ studied the notion of intuitionist fuzzy PMS ideals under homomorphism and Cartesian product and investigated several related properties.

A wide variety of human decision-making is based on double-sided or bipolar judgmental thinking on a positive side and a negative side, for instance, cooperation and competition, friendship and hostility, common interests and conflict interests, effect and side effect, likelihood and unlikelihood, feed forward and feedback. The notion of bipolar fuzzy sets (YinYang bipolar fuzzy sets) was introduced by Zhang ^11^ as a generaztion of fuzzy set.

Consider a bipolar fuzzy set, as follows:

Frog’s prey = {(mosquito, 1, 0), (dragon fly, 0.4, 0), (turtle, 0, 0), (snake, 0, −1)}.

We can see that membership degree 0 and non-membership degree 0 of turtle mean that frog never hunts turtle and turtle never hunts frog. While membership degree 0 and non-membership degree –1 of snake mean that frog never hunts snake but snake always hunts frog. Here, the implicit counter-property is “predator of frog,” which created the difference between fuzzy set and bipolar fuzzy set of frog’s prey.

Motivated by this, we introduced the notion of a bipolar fuzzy pseudo-UP ideal of pseudo-UP algebra and proved some results. We present several results related to the bipolar fuzzy pseudo-UP ideal in pseudo-UP algebras. We investigate the properties of the homomorphic image and preimage of bipolar fuzzy pseudo-UP ideals and demonstrate that both the homomorphic image and preimage of a bipolar fuzzy pseudo-UP ideal are themselves bipolar fuzzy pseudo-UP ideals. Furthermore, the Cartesian product of bipolar fuzzy pseudo-UP ideals of pseudo-UP algebras is introduced, and several properties are investigated.

## Preliminaries

Definition 2.1. [Ref. 12] A pseudo-UP algebra is algebra ( *X*, ·, _*_, 0) of type (2, 2, 0) which satisfies the following axioms: for any *x*, *y*, and *z* ∈ *X*

1)
$\left(y\cdot z\right)\cdot \left(\left(x\cdot y\right)*\left(x\cdot z\right)\right)=0\;\text{and}\;\left(y^{*}z\right)*\left(\left(x^{*}y\right)\cdot \left(x^{*}z\right)\right)=0$ ,
2)
$x\cdot y=0=y\cdot x\;\text{and}\;x^{*}y=0=y^{*}x\Rightarrow x=y$ ,
3)
$\left(y\cdot 0\right)*x=x\;\text{and}\;\left(y^{*}0\right)\cdot x=x$ ,
4) *x* ≤ *y* if and only if *x* · *y* = 0 and *x* ≤ *y* if and only if *x* * *y* = 0.

Proposition 2.2. [Ref. 12] In a pseudo-UP algebra *X* the following holds: for any *x*, *y* ∈ *X*

1)
x≤y⋅x and
2)
$x\leq y^{*}x$ .

Definition 2.3. [Ref. 2] A pseudo-UP ideal of *X* is a nonempty subset *J* of a pseudo-UP algebra *X*

That has the following: for each *x*, *y*, *z* ∈ *X.*

1)
0∈J ,
2)
$x\cdot \left(y^{*}z\right)\in J\;\text{and}\;y\in J\Rightarrow x\cdot z\in J$ and
3)
$x^{*}\left(y\cdot z\right)\in J\;\text{and}\;y\in J\Rightarrow x^{*}z\in J$ .

Lemma 2.4. [Ref. 2] In a pseudo-UP algebra *X* the following holds, for each *x* ∈ *X*,

1) *x* · 0 = 0 and *x* * 0 = 0,
2) 0 · *x* = *x* and 0 * *x* = *x* and
3) *x* · *x* = 0 and *x* * *x* = 0.

Definition 2.5. [Ref. 5] A fuzzy subset *λ* of a pseudo-UP algebras of *X* is called fuzzy pseudo-UP ideal of *X* if and only if it fulfill the following axioms: for any *x, y, z* ∈ *X*

1)
λ(0)≥λ(x) ,
2)
$\lambda \left(x\cdot y\right)\geq \text{min}\left\{\lambda \left(x\cdot \left(y^{*}z\right)\right),\lambda \left(y\right)\right\}$ and
3)
$\lambda \left(x^{*}y\right)\geq \text{min}\left\{\lambda \left(x^{*}\left(y\cdot z\right)\right),\lambda \left(y\right)\right\}$ .

Definition 2.6. [Ref. 8] Let *X* be the universe of discourse. A bipolar fuzzy set *λ* in *X* is an object having the form

$$\lambda =\left\{\left(x,\lambda^{-}\left(x\right),\lambda^{+}\left(x\right)\right):x\in X\right\}$$

Where *λ* ^−^: *X*→ [−1, 0] and *λ* ^+^: *X* → [0, 1] are mappings. Form this paper we use the symbol

*λ* = ( *X*; *λ* ^−^, *λ* ^+^) for the bipolar valued fuzzy set *λ* = {( *x*, *λ* ^−^( *x*), λ ^+^( *x*)): *x* ∈ *X*}, and use the notion of bipolar fuzzy sets instead of the notion of bipolar valued fuzzy sets.

Definition 2.7. [Ref. 13] A bipolar fuzzy set *λ* = ( *X*; *λ* ^+^, *λ* ^−^) in a set *X* with the positive membership

*λ* ^+^: *X* → [0, 1] and negative membership *λ* ^−^: *X* → [−1, 0] is indicated to have Sup-Inf property, if for any subset *T* of *X*, there exists *x*_0_ ∈ *T* such that $\lambda^{+}\left(x_{0}\right)=\underset{t\in T}{\sup}\lambda^{+}\left(t\right)$ and $\lambda^{-}\left(x_{0}\right)=\underset{t\in T}{\inf}\lambda^{-}\left(t\right)$

Lemma 2.8. Let λ = ( *X*, λ ^+^, λ ^−^) be bipolar fuzzy set in *X.* Then the following statements hold for any x, y ∈ *X.*

1)
$1-\text{max}\left\{\lambda^{+}\left(x\right),\lambda^{+}\left(y\right)\right\}=\text{min}\left\{1-\lambda^{+}\left(x\right),1-\lambda^{+}\left(y\right)\right\}$ ,
2)
$1-\text{min}\left\{\lambda^{+}\left(x\right),\lambda^{+}\left(y\right)\right\}=\text{max}\left\{1-\lambda^{+}\left(x\right),1-\lambda^{+}\left(y\right)\right\}$ ,
3)
$-1-\text{max}\left\{\lambda^{-}\left(x\right),\lambda^{-}\left(y\right)\right\}=\text{min}\left\{-1-\lambda^{-}\left(x\right),-1-\lambda^{-}\left(y\right)\right\}$ and
4)
$-1-\text{min}\left\{\lambda^{-}\left(x\right),\lambda^{-}\left(y\right)\right\}=\text{max}\left\{-1-\lambda^{-}\left(x\right),-1-\lambda^{-}\left(y\right)\right\}$ .

Definition 2.9. [Ref. 14] Let *f*: *X* → *Y* be a homomorphism from a set *X* onto a set *Y* and let *λ* = ( *X*; *λ* ^−^, *λ* ^+^) be a bipolar fuzzy set of *X* and *σ* = ( *Y*; *σ* ^−^, *σ* ^+^) be two bipolar fuzzy set of *Y*, then the homomorphic image *f* ( *λ*) is *f* ( *λ*) = ( *f* ( *λ* ^−^), *f* ( *λ* ^+^)) defined as for all *y* ∈ *Y.*

And

$$f\left(\lambda^{-}\right)\left(x\right)=\inf\lambda^{-}\left(x\right)/x\in f^{-1}\left(y\right)\;\text{if}\;f^{-1}\left(y\right)\neq Ø$$

$$f\left(\lambda^{+}\right)\left(x\right)=\sup\;\lambda^{+}\left(x\right)/x\in f^{-1}\left(y\right)\;\text{if}\;f^{-1}\left(y\right)\neq Ø.$$

The pre-image *f* ^−1^ ( *σ*) of *σ* under f is a bipolar set defined as *f* ^−1^ ( *σ* ^−^) ( *x*) = *σ* ^−^ (f(x)) and *f* ^−1^ ( *σ* ^+^) ( *x*)) = *σ* ^+^ ( *f*( *x*)), for all *x* ∈ *X.*

Definition 2.10. [Ref. 15] Let *λ* and *σ* are any two bipolar fuzz set of *X* and *Y* respectively. The Cartesian product of *λ* and *σ* is defined as *λ* × *σ* = ( *X* × *Y*, *λ* ^+^ × *σ* ^+^, *λ* ^−^ × *σ* ^−^) with *λ* ^+^ × *σ* ^+^ ( *x*, *y*) = $\min\left\{\lambda^{+}\left(x\right),\sigma^{+}\left(y\right)\right\}$ and $\lambda^{-}\times \sigma^{-}\left(x,y\right)=\max\left\{\lambda^{-}\left(x\right),\sigma^{-}\left(y\right)\right\}$ where $\lambda^{+}\times \sigma^{+}$ : X×Y→[0,1] and $\lambda^{-}\times \sigma^{-}$ : X×Y→[−1,0] , ∀(x,y)∈X×Y .

## Bipolar fuzzy pseudo-UP ideal

In this section, we introduce the concept of a bipolar fuzzy pseudo-UP ideal in pseudo-UP algebras and examine several important properties associated with bipolar fuzzy pseudo-UP ideals.

Definition 3.1. A bipolar fuzzy set *λ* = ( *X*, *λ* ^+^, *λ* ^−^) in *X* is called bipolar fuzzy pseudo-UP ideal of *X* if it satisfies the following conditions for all x, y, z ∈ *X.*

i) *λ* ^+^(0) ≥ *λ* ^+^( *x*) and *λ* ^−^(0) ≤ *λ* ^−^( *x*),
ii) *λ* ^+^( *x* · *z*) ≥ min { *λ* ^+^( *x* · ( *y* * *z*)), *λ* ^+^( *y*)},
iii) *λ* ^+^( *x* * *z*) ≥ min { *λ* ^+^( *x* * ( *y* · *z*)), *λ* ^+^( *y*)},
iv) *λ* ^−^( *x* · *z*) ≤ max { *λ* ^−^( *x* · ( *y* * *z*)), *λ* ^−^( *y*)},
v) *λ* ^−^( *x* * *z*) ≤ max { *λ* ^−^( *x* * ( *y* · *z*)), *λ* ^−^( *y*)}.

Example 3.2. Let *X* = {0, *a*, *b*, *c*} be a set with binary operations “·” and “*” defined by the following cayley Table 1

Clearly, ( *X*, ·, *, 0) is pseudo-UP algebra. We define a bipolar fuzzy pseudo-UP ideal of *X* as follows.

**Table:** 

*X*	0	a	b	c
*λ ^+^*	0.8	0.6	0.5	0.4
*λ* ^−^	-0.7	-0.5	-0.4	-0.4

Then, *λ* is a bipolar fuzzy pseudo-UP ideal of *X.*

Theorem 3.3. Let *λ* = ( *X*, *λ* ^+^, *λ* ^−^) be a bipolar fuzzy pseudo-UP ideal of a pseudo-UP algebra *X* with *y* ≤ *z*, for any *y*, *z* ∈ *X*, then *λ* ^+^( *y*) ≥ *λ* ^+^( *z*) and *λ* ^−^( *y*) ≤ *λ* ^−^( *z*), that is *λ* ^+^ is order reversing and *λ* ^−^ is order preserving.

Proof. Let *λ* = ( *X*, *λ* ^+^, *λ* ^−^) be a bipolar fuzzy pseudo-UP ideal of pseudo-UP algebra *X* such that *y* ≤ *z*, for all y, *z* ∈ *X.* Then by the binary relation ≤ define in *X*, we have *z* · *y* = 0 and *z* * *y* = 0.

$$\begin{array}{l} \mathrm{Now},\;\lambda^{+}\left(y\right)=\lambda^{+}\left(0\cdot y\right),\text{by Lemma}\;2.4\\ \;\geq \text{min}\left\{\lambda^{+}\left(0\cdot \left(z^{*}y\right)\right),\lambda^{+}\left(z\right)\right\},\left(\text{by Definition}\;3.1\right)\\ \;=\text{min}\left\{\lambda^{+}\left(z^{*}\;y\right),\lambda^{+}\left(z\right)\right\},\left(\text{by Lemma}\;2.4\right)\\ \;=\text{min}\left\{\lambda^{+}\left(0\right),\lambda^{+}\left(z\right)\right\},\left(\text{since}\;y\leq z\Rightarrow z^{*}y=0\right)\\ \;=\lambda^{+}\left(z\right).\\ \;\Rightarrow \lambda^{+}\left(y\right)\geq \lambda^{+}\left(z\right). \end{array}$$

$$\begin{array}{l} \lambda^{+}\left(y\right)=\lambda^{+}\left(0^{*}y\right),\text{by Lemma}\;2.4\\ \;\geq \text{min}\left\{\lambda^{+}\left(0^{*}\left(z\cdot y\right)\right),\lambda^{+}\left(z\right)\right\},\left(\text{by Definition}\;3.1\right)\\ \;=\text{min}\left\{\lambda^{+}\left(z\cdot y\right),\lambda^{+}\left(z\right)\right\},\left(\text{by Lemma}\;2.4\right)\\ \;=\text{min}\left\{\lambda^{+}\left(0\right),\lambda^{+}\left(z\right)\right\},\left(\text{since}\;y\leq z\Rightarrow z\cdot y=0\right)\\ \;=\lambda^{+}\left(z\right).\Rightarrow \lambda^{+}\left(y\right)\geq \lambda^{+}\left(z\right),\text{and} \end{array}$$

$$\begin{array}{l} \lambda^{-}\left(y\right)=\lambda^{-}\left(0\cdot y\right),\left(\text{by Lemma}\;2.4\right)\\ \;\leq \text{max}\left\{\lambda^{-}\left(0\cdot \left(z^{*}y\right)\right),\lambda^{-}\left(z\right)\right\},\left(\text{by Definition}\;3.1\right)\\ \;=\text{max}\left\{\lambda^{-}\left(z^{*}y\right),\lambda^{-}\left(z\right)\right\},\left(\text{by Lemma}\;2.4\right)\\ \;=\text{max}\left\{\lambda^{-}\left(0\right),\lambda^{-}\left(z\right)\right\},\left(\text{since}\;y\leq z\Rightarrow z^{*}y=0\right)\\ \;=\lambda^{-}\left(z\right).\\ \;\Rightarrow \lambda^{-}\left(y\right)\leq \lambda^{-}\left(z\right). \end{array}$$

$$\begin{array}{l} \lambda^{-}\left(y\right)=\lambda^{-}\left(0^{*}y\right),\left(\text{by Lemma}\;2.4\right)\\ \;\leq \text{max}\left\{\lambda^{-}\left(0^{*}\left(z\cdot y\right)\right),\lambda^{-}\left(z\right)\right\},\left(\text{by Definition}\;3.1\right)\\ \;=\text{max}\left\{\lambda^{-}\left(z\cdot y\right),\lambda^{-}\left(z\right)\right\},\left(\text{by Lemma}\;2.4\right)\\ \;=\text{max}\left\{\lambda^{-}\left(0\right),\lambda^{-}\left(z\right)\right\},\left(\text{since}\;y\leq z\Rightarrow z\cdot y=0\right)\\ \;=\lambda^{-}\left(z\right).\\ \;\Rightarrow \lambda^{-}\left(y\right)\leq \lambda^{-}\left(z\right). \end{array}$$

Hence, *λ* ^+^( *y*) ≥ *λ* ^+^ ( *z*) and *λ* ^−^( *y*) ≤ *λ* ^−^ ( *z*). *□*

Theorem 3.4. Every bipolar fuzzy pseudo-UP ideal *λ* = ( *X*, *λ* ^+^, *λ* ^−^) of *X* is a bipolar fuzzy pseudo- UP subalgebra of *X.*

Proof. Suppose *λ* is a fuzzy pseudo-UP ideal of *X.* Then,

$$\begin{array}{l} \lambda^{+}\left(x\cdot y\right)\geq \text{min}\left\{\lambda^{+}\left(x\cdot \left(y^{*}y\right)\right),\lambda^{+}\left(y\right)\right\},\left(\text{by Definition}\;3.1\right)\\ \;=\text{min}\left\{\lambda^{+}\left(x\cdot 0\right),\lambda^{+}\left(y\right)\right\},\left(\text{by Lemma}\;2.4\right)\\ \;=\text{min}\left\{\lambda^{+}\left(0\right),\lambda^{+}\left(y\right)\right\},\left(\text{by Lemma}\;2.4\right)\\ \;\geq \text{min}\left\{\lambda^{+}\left(x\right),\lambda^{+}\left(y\right)\right\},\left(\text{since}\;\lambda^{+}\left(0\right)\geq \lambda^{+}\left(x\right)\right)\\ \;\Rightarrow \lambda^{+}\left(x\cdot y\right)\geq \text{min}\left\{\lambda^{+}\left(x\right),\lambda^{+}\left(y\right)\right\}. \end{array}$$

$$\begin{array}{l} \lambda^{+}\left(x^{*}y\right)\geq \text{min}\left\{\lambda^{+}\left(x^{*}\left(y\cdot y\right)\right),\lambda^{+}\left(y\right)\right\},\left(\text{by Definition}\;3.1\right)\\ \;=\text{min}\left\{\lambda^{+}\left(x^{*}0\right),\lambda^{+}\left(y\right)\right\},\left(\text{by Proposition}\;2.2\right)\\ \;=\text{min}\left\{\lambda^{+}\left(0\right),\lambda^{+}\left(y\right)\right\},\left(\text{by Lemma}\;2.4\right)\\ \;\geq \text{min}\left\{\lambda^{+}\left(x\right),\lambda^{+}\left(y\right)\right\}\left(\text{since}\;\lambda^{+}\left(0\right)\geq \lambda^{+}\left(x\right)\right).\\ \;\Rightarrow \lambda^{+}\left(x^{*}y\right)\geq \text{min}\left\{\lambda^{+}\left(x\right),\lambda^{+}\left(y\right)\right\},\text{and} \end{array}$$

$$\begin{array}{l} \lambda^{-}\left(x\cdot y\right)\leq \text{max}\left\{\lambda^{-}\left(x\cdot \left(y^{*}\;y\right)\right),\lambda^{-}\left(y)\right)\right\},\left(\text{by Definition}\;3.1\right)\\ \;=\text{max}\left\{\lambda^{-}\left(x\cdot 0\right),\lambda^{-}\left(y\right)\right\},\left(\text{by Lemma}\;2.4\right)\\ \;=\text{max}\left\{\lambda^{-}\left(0\right),\lambda^{-}\left(y\right)\right\},\left(\text{by Lemma}\;2.4\right)\\ \;\leq \text{max}\left\{\lambda^{-}\left(x\right),\lambda^{-}\left(y\right)\right\}.\\ \;\Rightarrow \lambda^{-}\left(x\cdot y\right)\leq \text{max}\left\{\lambda^{-}\left(x\right),\lambda^{-}\left(y\right)\right\}. \end{array}$$

$$\begin{array}{l} \text{Finally},\;\lambda^{-}\left(x^{*}y\right)\leq \text{max}\left\{\lambda^{-}\left(x^{*}\left(y\geq y\right)\right),\lambda^{-}\left(y\right)\right\},\left(\text{by Definition}\;3.1\right)\\ \;=\text{max}\left\{\lambda^{-}\left(x^{*}0\right),\lambda^{-}\left(y\right)\right\},\left(\text{by Proposition}\;2.2\right)\\ \;=\text{max}\left\{\lambda^{-}\left(0\right),\lambda^{-}\left(y\right)\right\},\left(\text{by Lemma}\;2.4\right)\\ \;\leq \text{max}\left\{\lambda^{-}\left(x\right),\lambda^{-}\left(y\right)\right\}.\\ \;\Rightarrow \lambda^{-}\left(x^{*}y\right)\leq \text{max}\left\{\lambda^{-}\left(x\right),\lambda^{-}\left(y\right)\right\}. \end{array}$$

Thus, *λ* = ( *X*, *λ* ^+^, *λ* ^−^) is a bipolar fuzzy pseudo-UP subalgebra of pseudo-UP algebra *X.* □

Remark 3.5. The converse is may not be true.

Example 3.6. Let *X* = {0, 1, 2, and 3} be a set with a binary operations “·” and “*” defined by the following cayley Table 2.

Then, ( *X*, ·, *, 0) is a pseudo-UP algebra. We define a bipolar fuzzy set *λ* = ( *X*; *λ* ^+^, *λ* ^−^) in *X* as follows:

**Table:** 

*X*	0	1	2	3
*λ* ^+^	0.8	0.4	0.2	0.1
*λ*−	-0.9	-0.5	-0.3	-0.2

Then *λ* = ( *X*; *λ* ^+^, *λ* ^−^) is a bipolar fuzzy pseudo-UP subalgebra of *X*, but it is not a bipolar fuzzy pseudo-UP ideal of *X.* Indeed,

*λ* ^+^(0 · 2) ≥ min { *λ* ^+^(0 · (1 * 2)), *λ* ^+^(1)} = *λ* ^+^(2) ≥ min { *λ* ^+^(0 · 1), *λ* ^+^(1)} implies that 0.2 ≥ 0.4 which is contradict to Definition 3.1. And similarly, *λ* ^−^(0 · 2) ≤ max { *λ* ^−^(0 · (1 * 2)), *λ* ^−^(1)} = *λ* ^−^(2) ≤ max { *λ* ^−^(0 · 1), *λ* ^−^(1)} implies that −0.3 ≤ −0.5 which is contradict to Definition 3.1.

Theorem 3.7. Let *λ* = ( *X*; *λ* ^+^, *λ* ^−^) be a bipolar fuzzy pseudo-UP ideal of a pseudo-UP algebra *X.* If the inequality *x* ≤ *y* · *z* and *x* ≤ *y* * *z* holds in *X* for all *x*, *y*, *z* ∈ *X*, the *λ* ^+^( *z*) ≥ min { *λ* ^+^(x), *λ* ^+^( *y*)} and *λ* ^−^( *z*) ≤ max { *λ* ^−^( *x*), *λ* ^−^( *y*)}.

Proof. Let *x*, *y*, *z* ∈ *X* such that *x* ≤ *y* * *z* and *x* ≤ *y* · *z.* Then by the binary relation “≤” defined in *X*, we have *x* · ( *y* * *z*) = 0 and *x* * ( *y* · *z*) = 0. By Definition 3.1, we have

$$\lambda^{+}\left(x\cdot z\right)\geq \text{min}\left\{\lambda^{+}\left(x\cdot \left(y^{*}z\right)\right),\lambda^{+}\left(y\right)\right\}$$ (3.1)

And by Theorem 3.4, we have

$$\lambda^{+}\left(z\right)=\lambda^{+}\left(0^{*}z\right)\geq \text{min}\left\{\lambda^{+}\left(0^{*}\left(x\cdot z\right)\right),\lambda^{+}\left(x\right)\right\}=\text{min}\left\{\lambda^{+}\left(x\cdot z\right),\lambda^{+}\left(x\right)\right\}$$ (3.2)

By (3.1) and Definition 3.1

$$\lambda^{+}\left(x\cdot z\right)\geq \text{min}\left\{\lambda^{+}\left(x^{*}\left(y\cdot z\right)\right),\lambda^{+}\left(y\right)\right\}=\text{min}\left\{\lambda^{+}\left(0\right),\lambda^{+}\left(y\right)\right\}$$ (3.3)

By (3.2) and (3.3), we have

Similarly,

$$\lambda^{+}\left(z\right)\geq \text{min}\left\{\lambda^{+}\left(x\cdot z\right),\lambda^{+}\left(x\right)\right\}\geq \text{min}\left\{\lambda^{+}\left(y\right),\lambda^{+}\left(x\right)\right\}=\text{min}\left\{\lambda^{+}\left(x\right),\lambda^{+}\left(y\right)\right\}.$$

$$\lambda^{-}\left(x\cdot z\right)\leq \text{max}\left\{\lambda^{-}\left(x\cdot \left(y^{*}z\right)\right),\lambda^{-}\left(y\right)\right\}$$ (3.4)

And by Theorem 3.4, we have

$$\lambda^{-}\left(z\right)=\lambda^{-}\left(0^{*}z\right)\leq \text{max}\left\{\lambda^{-}\left(0^{*}\left(x\cdot z\right)\right),\lambda^{-}\left(x\right)\right\}=\text{max}\left\{\lambda^{-}\left(x\cdot z\right),\lambda^{-}\left(x\right)\right\}$$ (3.5)

By (3.4) and Definition 3.1

$$\lambda^{-}\left(x\cdot z\right)\leq \text{max}\left\{\lambda^{-}\left(x^{*}\left(y\cdot z\right)\right),\lambda^{-}\left(y\right)\right\}=\text{max}\left\{\lambda^{-}\left(0\right),\lambda^{-}\left(y\right)\right\}$$ (3.6)

By (3.5) and (3.6), we have

$$\lambda^{-}\left(z\right)\leq \text{max}\left\{\lambda^{-}\left(x\cdot z\right),\lambda^{-}\left(x\right)\right\}\leq \text{max}\left\{\lambda^{-}\left(y\right),\lambda^{-}\left(x\right)\right\}=\text{max}\left\{\lambda^{-}\left(x\right),\lambda^{-}\left(y\right)\right\}.$$

Hence, $\lambda^{+}\left(z\right)\geq \text{min}\left\{\lambda^{+}\left(x\right),\lambda^{+}\left(y\right)\right\}\text{and}\lambda^{-}\left(z\right)\leq \text{max}\left\{\lambda^{-}\left(x\right),\lambda^{-}\left(y\right)\right\}.$ □

Theorem 3.8. The intersection of any two bipolar fuzzy pseudo-UP ideals of a pseudo-UP algebra *X* is also a bipolar fuzzy pseudo-UP ideal.

Proof. Let *λ* = ( *X*; *λ* ^+^, *λ* ^−^) and *η* = ( *X*; *η* ^+^, *η* ^−^) be two bipolar fuzzy pseudo-UP ideals of *X.* Then we claim that *λ* ∩ *η* is a bipolar fuzzy pseudo-UP ideal of *X.* Let *x*, *y*, *z* ∈ *X.* Then

$\lambda^{+}\cap \eta^{+}\left(0\right)=\text{min}\left\{\lambda^{+}\left(0\right),\eta^{+}\left(0\right)\right\}\geq \text{min}\left\{\lambda^{+}\left(x\right),\eta^{+}\left(x\right)\right\}=\lambda^{+}\cap \eta^{+}\left(x\right).$

And $\lambda^{-}\cap \eta^{-}\left(0\right)=\text{max}\left\{\lambda^{-}\left(0\right),\eta^{-}\left(0\right)\right\}\leq \text{max}\left\{\lambda^{-}\left(x\right),\eta^{-}\left(x\right)\right\}=\lambda^{-}\cap \eta^{-}\left(x\right).$

$$\begin{array}{l} \text{Also},\;\lambda^{+}\cap \eta^{+}\left(x\cdot z\right)=\text{min}\left\{\lambda^{+}\left(x\cdot z\right),\eta^{+}\left(x\cdot z\right)\right\}\\ \;\geq \text{min}\left\{\text{min}\left\{\lambda^{+}\left(x\cdot \left(y^{*}z\right)\right),\lambda^{+}\left(y\right)\right\},\text{min}\left\{\eta^{+}\left(x\cdot \left(y^{*}z\right)\right),\eta^{+}\left(y\right)\right\}\right\}\\ \;=\text{min}\left\{\text{min}\left\{\lambda^{+}\left(x\cdot \left(y^{*}z\right)\right),\eta^{+}\left(x\cdot \left(y^{*}z\right)\right)\right\},\text{min}\left\{\lambda^{+}\left(y\right),\eta^{+}\left(y\right)\right\}\right\}\\ \;=\text{min}\left\{\lambda^{+}\cap \eta^{+}\left(x\cdot \left(y^{*}z\right)\right),\lambda^{+}\cap \eta^{+}\left(y\right)\right\},\text{and} \end{array}$$

$$\begin{array}{l} \lambda^{+}\cap \eta^{+}\left(x^{*}z\right)=\text{min}\left\{\lambda^{+}\left(x^{*}z\right),\eta^{+}\left(x^{*}z\right)\right\}\\ \;\geq \text{min}\left\{\text{min}\left\{\lambda^{+}\left(x^{*}\left(y\cdot z\right)\right),\lambda^{+}\left(y\right)\right\},\text{min}\left\{\eta^{+}\left(x^{*}\left(y\cdot z\right)\right),\eta^{+}\left(y\right)\right\}\right\}\\ \;=\text{min}\left\{\text{min}\left\{\lambda^{+}\left(x^{*}\left(y\cdot z\right)\right),\eta^{+}\left(x^{*}\left(y\cdot z\right)\right)\right\},\text{min}\left\{\lambda^{+}\left(y\right),\eta^{+}\left(y\right)\right\}\right\}\\ \;=\text{min}\left\{\lambda^{+}\cap \eta^{+}\left(x^{*}\left(y\cdot z\right)\right),\lambda^{+}\cap \eta^{+}\left(y\right)\right\}. \end{array}$$

$$\begin{array}{l} \text{Additionally},\;\lambda^{-}\cap \eta^{-}\left(x\cdot z\right)=\text{max}\left\{\lambda^{-}\left(x\cdot z\right),\eta^{-}\left(x\cdot z\right)\right\}\\ \;\leq \text{max}\left\{\text{max}\left\{\lambda^{-}\left(x\cdot \left(y^{*}z\right)\right),\lambda^{-}\left(y\right)\right\},\text{max}\left\{\eta^{-}\left(x\cdot \left(y^{*}z\right)\right),\eta^{-}\left(y\right)\right\}\right\}\\ \;=\text{max}\left\{\text{max}\left\{\lambda^{-}\left(x\cdot \left(y^{*}z\right)\right),\eta^{-}\left(x\cdot \left(y^{*}z\right)\right)\right\},\text{max}\left\{\lambda^{-}\left(y\right),\eta^{-}\left(y\right)\right\}\right\}\\ \;=\text{max}\left\{\lambda^{-}\cap \eta^{-}\left(x\cdot \left(y^{*}z\right)\right),\lambda^{-}\cap \eta^{-}\left(y\right)\right\}. \end{array}$$

$$\begin{array}{l} \text{And}\;\lambda^{-}\cap \eta^{-}\left(x^{*}z\right)=\text{max}\left\{\lambda^{-}\left(x^{*}z\right),\eta^{-}\left(x^{*}z\right)\right\}\\ \;\leq \text{max}\left\{\text{max}\left\{\lambda^{-}\left(x^{*}\left(y\cdot z\right)\right),\lambda^{-}\left(y\right)\right\},\text{max}\left\{\eta^{-}\left(x^{*}\left(y\cdot z\right)\right),\eta^{-}\left(y\right)\right\}\right\}\\ \;=\text{max}\left\{\text{max}\left\{\lambda^{-}\left(x^{*}\left(y\cdot z\right)\right),\eta^{-}\left(x^{*}\left(y\cdot z\right)\right)\right\},\text{max}\left\{\lambda^{-}\left(y\right),\eta^{-}\left(y\right)\right\}\right\}\\ \;=\text{max}\left\{\lambda^{-}\cap \eta^{-}\left(x^{*}\left(y\cdot z\right)\right),\lambda^{-}\cap \eta^{-}\left(y\right)\right\}. \end{array}$$

Hence, *λ* ∩ *η* is a bipolar fuzzy pseudo-UP ideal of *X.* □

Corollary 3.9. The intersection of any set of bipolar fuzzy pseudo-UP ideals in a pseudo-UP algebra *X* is also a bipolar fuzzy pseudo-UP ideal.

Remark 3.10. The union of two bipolar fuzzy pseudo-UP ideals may not be bipolar fuzzy pseudo-UP ideal.

Example 3.11. Let *X* = {0, 1, 2, and 3} be a set with a binary operation “·” and “*” defined by the following cayley Table 3.

Clearly, ( *X*, ·, *, 0) is a pseudo-UP algebra. We define a fuzzy set *λ* ^+^: *X* → [0, 1] as follows, *λ* ^+^(0) = 1, *λ* ^+^(1) = 0.6, *λ* ^+^(2) = 0.4, *λ* ^+^(3) = 0.3 and we define a fuzzy set *λ* ^−^: *X* → [−1, 0] as follows *λ* ^−^(0) = −1, *λ* ^−^(1) = −0.5, *λ* ^−^(2) = −0.6, *λ* ^−^(3) = −0.4 and a fuzzy set *η* ^+^: *X* → [0, 1] define as follows, *η* ^+^(0) = 1, *η* ^+^(1) = 0.4, *η* ^+^(2) = 0.5, *η* ^+^(3) = 0.3. and *η* ^−^: *X* → [−1, 0] define as follows *η* ^−^(0) = −0.9, *η* ^−^(1) = −0.5, *η* ^−^(2) = −0.3, *η* ^−^(3) = −0.2. Now, ( *λ* ^+^ ∪ *η* ^+^)(1 · 3) = max { *λ* ^+^(1 · 3), *η* ^+^(1 · 3)} = max { *λ* ^+^(3), *η* ^+^(3)} = max{0.3, 0.3} = 0.3.

Hence, ( *λ* ^+^ ∪ *η* ^+^) (1 · 3) = 0.3=.....................................(*).

$$\begin{array}{l} \left(\lambda^{+}\cup \eta^{+}\right)\left(1\cdot 3\right)=\text{max}\left\{\lambda^{+}\left(1\cdot 3\right),\eta^{+}\left(1\cdot 3\right)\right\}\\ \;\geq \text{max}\left\{\text{min}\left\{\lambda^{+}\left(1\cdot \left(2^{*}3\right)\right),\lambda^{+}\left(2\right)\right\},\text{min}\left\{\eta^{+}\left(1\cdot \left(2^{*}3\right)\right),\eta^{+}\left(2\right)\right\}\right\}\\ \;=\text{max}\left\{\text{min}\left\{\lambda^{+}\left(1\cdot 0\right),\eta^{+}\left(1\cdot 0\right)\right\},\text{min}\left\{\lambda^{+}\left(2\right),\eta^{+}\left(2\right)\right\}\right\}\\ \;=\text{max}\left\{\text{min}\left\{\lambda^{+}\left(0\right),\eta^{+}\left(0\right)\right\},\text{min}\left\{\lambda^{+}\left(2\right),\eta^{+}\left(2\right)\right\}\right\}\\ \;=\text{max}\left\{\text{min}\left\{1,1\right\},\text{min}\left\{0.4,0.5\right\}\right\}=\text{max}\left\{1,0.5\right\}=1. \end{array}$$

From (*) we get 0.3 ≥ 1 which is contradict to Definition 3.1.

*And* $\left(\lambda^{-}\cup \eta^{-}\right)\left(1\cdot 3\right)=\text{min}\left\{\lambda^{-}\left(1\cdot 3\right),\eta^{-}\left(1\cdot 3\right)\right\}=\text{min}\left\{\lambda^{-}\left(3\right),\eta^{-}\left(3\right)\right\}=\text{min}\left\{-0.4,-0.2\right\}=-0.4\left({}^{*}*\right)$ .

$$\begin{array}{l} \left(\lambda^{-}\cup \eta^{-}\right)\left(1\cdot 3\right)=\text{min}\left\{\lambda^{-}\left(1\cdot 3\right),\eta^{-}\left(1\cdot 3\right)\right\}\\ \;\leq \text{min}\left\{\text{max}\left\{\lambda^{-}\left(1\cdot \left(2^{*}3\right)\right),\lambda^{-}\left(2\right)\right\},\text{max}\left\{\eta^{-}\left(1\cdot \left(2^{*}3\right)\right),\eta^{-}\left(2\right)\right\}\right\}\\ \;=\text{min}\left\{\text{max}\left\{\lambda^{-}\left(1\cdot 0\right),\eta^{-}\left(1\cdot 0\right)\right\},\text{max}\left\{\lambda^{-}\left(2\right),\eta^{-}\left(2\right)\right\}\right\}\\ \;=\text{min}\left\{\text{max}\left\{\lambda^{-}\left(0\right),\eta^{-}\left(0\right)\right\},\text{max}\left\{\lambda^{-}\left(2\right),\eta^{-}\left(2\right)\right\}\right\}\\ \;=\text{min}\left\{\text{max}\left\{-1,-0.9\right\},\text{max}\left\{-0.6,-0.3\right\}\right\}=\text{min}\left\{-0.9,-0.3\right\}=-0.9. \end{array}$$

From (**), we get −0.4 ≤ −0.9 which is contradict to Definition 3.1. This shows that the union of any two bipolar fuzzy pseudo-UP ideal of *X* may not be a bipolar fuzzy pseudo-UP ideal of *X.*

Theorem 3.12. A bipolar fuzzy set *λ* = ( *X*, *λ* ^+^, *λ* ^−^) in *X* is a bipolar fuzzy pseudo-UP ideal of *X*

If and only if the fuzzy subset *λ* ^+^ and ( *λ*−) ^c^ are fuzzy pseudo-UP ideals of *X.*

Proof. Let *λ* = ( *X*, *λ* ^+^, *λ* ^−^) be a bipolar fuzzy pseudo-UP algebra of *X.* We need to show that the fuzzy subset *λ* ^+^ and ( *λ*−) *^c^* are fuzzy pseudo-UP ideals of *X.* Clearly, *λ* ^+^ is a fuzzy pseudo-UP ideal of *X* follows from the fact that ( *X*, *λ* ^+^, *λ* ^−^) is a bipolar fuzzy pseudo-UP ideal of *X.* Now, it remains to show that ( *λ*−) *^c^* is a fuzzy pseudo-UP ideal of *X.* Let *x*, *y*, *z* ∈ *X*, then we have ( *λ*−) *^c^* (0) = −1 − *λ* ^−^ (0) ≥ −1 − *λ* ^−^( *x*) = ( *λ* ^−^) *^c^* ( *x*).

$$\begin{array}{l} \text{Next},\;{\left(\lambda -\right)}^{c}\left(x\cdot z\right)=-1-\lambda^{-}\left(x\cdot z\right)\geq -1-\text{max}\left\{\lambda^{-}\left(x\cdot \left(y^{*}z\right)\right),\lambda^{-}\left(y\right)\right\}\\ \;=\text{min}\left\{-1-\lambda^{-}\left(x\cdot \left(y^{*}z\right)\right),-1-\lambda^{-}\left(y\right)\right\}\;\left(\text{by Lemma}\right)2.8\left(3\right)\\ \;=\text{min}\left\{{\left(\lambda -\right)}^{c}\left(x\cdot \left(y^{*}z\right)\right),{\left(\lambda -\right)}^{c}\left(y\right)\right\}. \end{array}$$

$$\begin{array}{l} \text{Moreover},\;{\left(\lambda -\right)}^{c}\left(x^{*}z\right)=-1-\lambda^{-}\left(x^{*}z\right)\geq -1-\text{max}\left\{\lambda^{-}\left(x^{*}\left(y\cdot z\right)\right),\lambda^{-}\left(y\right)\right\}\\ \;=\text{min}\left\{-1-\lambda^{-}\left(x^{*}\left(y\cdot z\right)\right),-1-\lambda^{-}\left(y\right)\right\}\;\left(\text{by Lemma}\right)2.8\left(3\right)\\ \;=\text{min}\left\{{\left(\lambda -\right)}^{c}\left(x^{*}\left(y\cdot z\right)\right),{\left(\lambda -\right)}^{c}\left(y\right)\right\}. \end{array}$$

Hence, ( *λ*−) *^c^* is a fuzzy pseudo-UP ideal of *X.*

Conversely, assume that *λ* ^+^ and ( *λ*−) *^c^* is fuzzy pseudo-UP ideal of *X.* Then for every *x*, *y*, *z* ∈ *X*, we get *λ* ^+^ (0) ≥ *λ* ^+^( *x*) and ( *λ*−) *^c^* (0) ≥ ( *λ*−) *^c^* ( *x*), by Definition 3.1

Now, ( *λ*−) *^c^* (0) ≥ ( *λ*−) *^c^* ( *x*) implies that −1 − *λ* ^−^ (0) ≥ −1 − *λ* ^−^( *x*), then *λ* ^−^ (0) ≤ *λ* ^−^( *x*). Now it is suffices to show that *λ* ^−^( *x*~ *z*) ≤ max { *λ* ^−^( *x*·( y* *z*)), *λ* ^−^( *y*)} and *λ* ^−^( x* *z*) ≤ max { *λ* ^−^( x*( *y* · *z*)), *λ* ^−^( *y*)}, for all *x*, *y*, *z* ∈ *X.*

$$\begin{array}{l} -1-\lambda^{-}\left(x\cdot z\right)={\left(\lambda -\right)}^{c}\left(x\cdot z\right)\geq \text{min}\left\{{\left(\lambda -\right)}^{c}\left(x\cdot \left(y^{*}z\right)\right),{\left(\lambda -\right)}^{c}\left(y\right)\right\}\\ \;=\text{min}\left\{-1-\lambda^{-}\left(x\cdot \left(y^{*}z\right)\right),-1-\lambda^{-}\left(y\right)\right\}\\ \;=-1-\text{max}\left\{\lambda^{-}\left(x\cdot \left(y^{*}z\right)\right),\lambda^{-}\left(y\right)\right\},\;\left(\text{by Lemma}\right)2.8\\ \;\Rightarrow \lambda^{-}\left(x\cdot z\right)\leq \text{max}\left\{\lambda^{-}\left(x\cdot \left(y^{*}z\right)\right),\lambda^{-}\left(y\right)\right\}. \end{array}$$

$$\begin{array}{l} \text{Finally},-1-\lambda^{-}\left(x^{*}z\right)={\left(\lambda^{-}\right)}^{c}\left(x^{*}z\right)\geq \text{min}\left\{{\left(\lambda^{-}\right)}^{c}x^{*}\left(y\cdot z\right),{\left(\lambda^{-}\right)}^{c}\left(y\right)\right\}\\ \;=\text{min}\left\{-1-\lambda^{-}\left(x^{*}\left(y\cdot z\right)\right),-1-\lambda^{-}\left(y\right)\right\}\\ \;=-1-\text{max}\left\{\lambda^{-}\left(x^{*}\left(y\cdot z\right)\right),\lambda^{-}\left(y\right)\right\},\\ \;\Rightarrow \lambda^{-}\left(x^{*}z\right)\leq \text{max}\left\{\lambda^{-}\left(x^{*}\left(y\cdot z\right)\right),\lambda^{-}\left(y\right)\right\}.\;\left(\text{by Lemma}\right)2.8 \end{array}$$

Hence, *λ* = ( *X*, *λ* ^+^, *λ* ^−^) is a bipolar fuzzy pseudo-UP ideal of *X.* □

Corollary 3.13. If *λ* ^+^ is a fuzzy pseudo-UP ideal of *X*, then *λ* = ( *X*, *λ* ^+^, (( *λ* ^+^) *^c^*) is a bipolar fuzzy pseudo-UP ideal of *X.*

Proof. Suppose *λ* ^+^ is a fuzzy pseudo-UP ideal of *X.* Then we need to show that *λ* = ( *X*, *λ* ^+^, ( *λ*−) *^c^*) is a bipolar fuzzy pseudo-UP ideal of *X.* Since *λ* ^+^ is a fuzzy pseudo-UP ideal of *X*, it follows that *λ* ^+^ (0) ≥ *λ* ^+^( *x*), *λ* ^+^( *x*· *z*) ≥ min { *λ* ^+^( *x*· ( *y* * *z*)), *λ* ^+^( *y*)} and *λ* ^+^( x* *z*) ≥ min { *λ* ^+^( x*( *y* · *z*)), *λ* ^+^( *y*)}, for all *x*, *y*, *z* ∈ *X.* Then it is enough to show that ( *λ* ^+^) *^c^* (0) ≥ ( *λ* ^+^) *^c^* ( *x*), ( *λ* ^+^) *^c^* ( *x* · *z*) ≥ min{( *λ* ^+^) *^c^* ( *x* · ( *y* * *z*)), ( *λ* ^+^) *^c^* ( *y*)} and ( *λ* ^+^) *^c^* ( *x* * *z*) ≥ min{( *λ* ^+^) *^c^* ( *x* * ( *y* · *z*)), ( *λ* ^+^) *^c^* ( *y*)}.

$$\mathrm{Now},{\left(\lambda^{+}\right)}^{c}\left(0\right)=1-\lambda^{+}\left(0\right)\leq 1-\lambda^{+}\left(x\right)={\left(\lambda^{+}\right)}^{c}\left(x\right)\Rightarrow {\left(\lambda^{+}\right)}^{c}\left(0\right)\leq {\left(\lambda^{+}\right)}^{c}\left(x\right)\;\text{and}$$

$$\begin{array}{l} {\left(\lambda^{+}\right)}^{c}\left(x\cdot z\right)=1-\lambda^{+}\left(x\cdot z\right)\leq 1-\text{min}\left\{\lambda^{+}\left(x\cdot \left(y^{*}z\right)\right),\lambda^{+}\left(y\right)\right\}\\ \;=\text{max}\left\{1-\lambda^{+}\left(x\cdot \left(y^{*}z\right)\right),1-\lambda^{+}\left(y\right)\right\}\\ \;=\text{max}\left\{{\left(\lambda^{+}\right)}^{c}\left(x\cdot \left(y^{*}z\right)\right),{\left(\lambda^{+}\right)}^{c}\left(y\right)\right\}.\\ \;\Rightarrow {\left(\lambda^{+}\right)}^{c}\left(x\cdot z\right)\leq \text{max}\left\{{\left(\lambda^{+}\right)}^{c}\left(x\cdot \left(y^{*}z\right)\right),{\left(\lambda^{+}\right)}^{c}\left(y\right)\right\}. \end{array}$$

$$\begin{array}{l} \text{Finally}\;{\left(\lambda^{+}\right)}^{c}\left(x^{*}z\right)=1-\lambda^{+}\left(x^{*}z\right)\leq 1-\text{min}\left\{\lambda^{+}\left(x^{*}\left(y.z\right)\right),\lambda^{+}\left(y\right)\right\}\\ \;=\text{max}\left\{1-\lambda^{+}\left(x^{*}\left(y.z\right)\right),1-\lambda^{+}\left(y\right)\right\}\\ \;=\text{max}\left\{{\left(\lambda^{+}\right)}^{c}\left(x^{*}\left(y.z\right)\right),{\left(\lambda^{+}\right)}^{c}\left(y\right)\right\}.\\ \;\Rightarrow {\left(\lambda^{+}\right)}^{c}\left(x\cdot z\right)\leq \text{max}\left\{{\left(\lambda^{+}\right)}^{c}\left(x^{*}\left(y.z\right)\right),{\left(\lambda^{+}\right)}^{c}\left(y\right)\right\}. \end{array}$$

Therefore, *λ* = ( *X*, *λ* ^+^, ( *λ* ^+^) *^c^*) is a bipolar fuzzy pseudo-UP ideal of *X.* □

Corollary 3.14. If ( *λ*−) *^c^* is a fuzzy pseudo-UP ideal of *X*, then *λ* = ( *X*, ( *λ* ^−^) *^c^*), *λ* ^−^ is a bipolar fuzzy pseudo-UP ideal of *X.*

Proof. Similar to Corollary 3.13.

Using those, corollary the following is obtained.

Theorem 3.15. A bipolar fuzzy set *λ* = ( *X*, *λ* ^+^, *λ* ^−^) of *X* is a bipolar fuzzy pseudo-UP ideal of *X* if and only if *λ* = ( *X*, *λ* ^+^, ( *λ* ^+^) *^c^*) and ♦ *λ* = ( *X*, ( *λ* ^−^) *^c^*, *λ* ^−^) are bipolar fuzzy pseudo-UP ideals of *X.*

Proof. Suppose *λ* = ( *X*, *λ* ^+^, *λ* ^−^) is bipolar fuzzy pseudo-UP ideal of *X*, then for any *x*, *y*, *z* ∈ *X*, we have *λ* ^+^(0) ≥ *λ* ^+^( *x*), *λ* ^+^( *x*· *z*) ≥ min { *λ* ^+^( *x*·( y* *z*)), *λ* ^+^( *y*)} and *λ* ^+^( x* *z*) ≥ min { *λ* ^+^( x*( *y*· *z*)), *λ* ^+^( *y*)}. Next we have to show that ( *λ* ^+^) *^c^* satisfies the conditions such that ( *λ* ^+^) *^c^* (0) ≤ ( *λ* ^+^) *^c^* ( *x*), ( *λ* ^+^) *^c^* ( *x*· *z*) ≤ max{( *λ* ^+^) *^c^* ( *x* · ( *y* * *z*)), ( *λ* ^+^) *^c^* ( *y*)} and ( *λ* ^+^) *^c^* ( *x* * *z*) ≤ max{( *λ* ^+^) *^c^* ( *x* * ( *y* · *z*)), ( *λ* ^+^) *^c^* ( *y*)}.

$$\mathrm{Now},\;{\left(\lambda^{+}\right)}^{c}\left(0\right)=1-\lambda^{+}\left(0\right)\leq 1-\lambda^{+}\left(x\right)={\left(\lambda^{+}\right)}^{c}\left(x\right)\Rightarrow {\left(\lambda^{+}\right)}^{c}\left(0\right)\leq {\left(\lambda^{+}\right)}^{c}\left(x\right).$$

$$\begin{array}{l} \text{Next},\;{\left(\lambda^{+}\right)}^{c}\left(x\cdot z\right)=1-\lambda^{+}\left(x\cdot z\right)\leq 1-\text{min}\left\{\lambda^{+}\left(x\cdot \left(y^{*}z\right)\right),\lambda^{+}\left(y\right)\right\}\\ \;=\text{max}\left\{1-\lambda^{+}\left(x\cdot \left(y^{*}z\right)\right),1-\lambda^{+}\left(y\right)\right\}\\ \;=\text{max}\left\{{\left(\lambda^{+}\right)}^{c}\left(x\cdot \left(y^{*}z\right)\right),{\left(\lambda^{+}\right)}^{c}\left(y\right)\right\}.\\ \;\Rightarrow {\left(\lambda^{+}\right)}^{c}\left(x\cdot z\right)\leq \text{max}\left\{{\left(\lambda^{+}\right)}^{c}\left(x\cdot \left(y^{*}z\right)\right),{\left(\lambda^{+}\right)}^{c}\left(y\right)\right\}. \end{array}$$

$$\begin{array}{l} \text{And also}\;{\left(\lambda^{+}\right)}^{c}\left(x^{*}z\right)=1-\lambda^{+}\left(x^{*}z\right)\leq 1-\text{min}\left\{\lambda^{+}\left(x^{*}\left(y.z\right)\right),\lambda^{+}\left(y\right)\right\}\\ \;=\text{max}\left\{1-\lambda^{+}\left(x^{*}\left(y.z\right)\right),1-\lambda^{+}\left(y\right)\right\}\\ \;=\text{max}\left\{{\left(\lambda^{+}\right)}^{c}\left(x^{*}\left(y.z\right)\right),{\left(\lambda^{+}\right)}^{c}\left(y\right)\right\}.\\ \;\Rightarrow {\left(\lambda^{+}\right)}^{c}\left(x^{*}z\right)\leq \text{max}\left\{{\left(\lambda^{+}\right)}^{c}\left(x^{*}\left(y.z\right)\right),{\left(\lambda^{+}\right)}^{c}\left(y\right)\right\}. \end{array}$$

Hence, *λ* is a bipolar fuzzy pseudo-UP ideal of *X.*

Also, for any *x*, *y*, *z* ∈ *X*, *λ* ^−^ (0) ≤ *λ* ^−^( *x*), *λ* ^−^( *x* · *z*) ≤ max { *λ* ^−^( *x* · ( *y* * *z*)), *λ* ^−^( *y*)} and *λ* ^−^( *x* * *z*) ≥ max { *λ* ^−^( *x* * ( *y* · *z*)), *λ* ^−^( *y*)}. Now we have to show that ( *λ* ^+^) ^*c*^ (0) ≥ ( *λ* ^+^) *^c^* ( *x*), ( *λ* ^+^) *^c^* ( *x* · *z*) ≥ min {( *λ* ^+^) *^c^* ( *x* · ( *y* * *z*)), ( *λ* ^+^) *^c^* ( *y*)} and ( *λ* ^+^) *^c^* ( *x* * *z*) ≤ min {( *λ* ^+^) *^c^* ( *x* * ( *y* · *z*)), ( *λ* ^+^) *^c^* ( *y*)}. So for any *x*, *y*, *z* ∈ *X*, we have ( *λ* ^-^) *^c^* (0) = −1 − *λ* ^−^(0) ≥ −1 − *λ* ^−^( *x*) = (λ ^-^) *^c^* ( *x*) ⇒ ( *λ* ^−^) *^c^* (0) ≥ ( *λ* ^−^) *^c^* ( *x*).

$$\begin{array}{l} \text{Next},\;{\left(\lambda^{-}\right)}^{c}\left((x\cdot z\right)=-1-\lambda^{-}\left(x\cdot z\right)\geq -1-\text{max}\left\{\lambda^{-}\left(x\cdot \left(y^{*}z\right)\right),\lambda^{-}\left(y\right)\right\}\\ \;=\text{min}\left\{-1-\lambda^{-}\left(x\cdot \left(y^{*}z\right)\right),-1-\lambda^{-}\left(y\right)\right\}\\ \;=\text{min}\left\{{\left((\lambda^{-}\right)}^{c}\left(x\cdot \left(y^{*}z\right)\right),{\left(\lambda^{-}\right)}^{c}\left(y\right)\right\}.\\ \;\Rightarrow {\left(\lambda^{-}\right)}^{c}\left(x\cdot z\right)\geq \text{min}\left\{{\left(\lambda^{-}\right)}^{c}\left(x\cdot \left(y^{*}z\right)\right),{\left(\lambda^{-}\right)}^{c}\left(y\right)\right\}. \end{array}$$

$$\begin{array}{l} \text{Finally},\;{\left(\lambda^{-}\right)}^{c}\left(x^{*}z\right)=-1-\lambda^{-}\left(x^{*}z\right)\geq -1-\text{max}\left\{\lambda^{-}\left(x^{*}\left(y\cdot z\right)\right),\lambda^{-}\left(y\right)\right\}\\ \;=\text{min}\left\{-1-\lambda^{-}\left(x^{*}\left(y\cdot z\right)\right),-1-\lambda^{-}\left(y\right)\right\}\\ \;=\text{min}\left\{{\left(\lambda^{-}\right)}^{c}\left(x^{*}\left(y\cdot z\right)\right),{\left(\lambda^{-}\right)}^{c}\left(y\right)\right\}.\\ \;\Rightarrow {\left(\lambda^{-}\right)}^{c}\left(x^{*}z\right)\geq \text{min}\left\{{\left(\lambda^{-}\right)}^{c}\left(x^{*}\left(y\cdot z\right)\right),{\left(\lambda^{-}\right)}^{c}\left(y\right)\right\}. \end{array}$$

Hence, ♦ *λ* is a bipolar fuzzy pseudo-UP ideal of *X.* The proof of the converse follows from Definition 3.1.□

Theorem 3.16. If *λ* = ( *X*, *λ* ^+^, *λ* ^−^) is a bipolar fuzzy pseudo-UP ideal of *X*, then *λ ^c^* is also a bipolar fuzzy pseudo-UP ideal of *X.*

Proof. Let *λ* = ( *X*, *λ* ^+^, *λ* ^−^) be a bipolar fuzzy pseudo-UP ideal of *X.* Then *λ* ^*c*^ = ( *X*, ( *λ* ^+^) ^*c*^ ( *λ* ^−^) ^*c*^), where ( *λ* ^+^) ^*c*^ (x) = 1 − *λ* ^+^( *x*) and ( *λ* ^−^) ^*c*^ ( *x*) = −1 − *λ* ^−^( *x*). Now, for any *x*, *y*, *z* ∈ *X*, we have ( *λ* ^−^) ^*c*^ (0) = −1− *λ* ^−^(0) ≥ −1− *λ* ^−^( *x*) = ( *λ* ^−^( *λ* ^−^) ^*c*^ ( *x*) ⇒ ( *λ* ^−^) ^*c*^ (0) ≥ ( *λ* ^−^) ^*c*^ ( *x*) and ( *λ* ^+^) ^*c*^ 0) = 1− *λ* ^+^(0) ≤ 1 − *λ* ^+^(x) = ( *λ* ^+^) ^*c*^ (x) ⇒ ( *λ* ^+^) ^*c*^ (0) ≤ ( *λ* ^+^) ^*c*^ (x).

$$\begin{array}{l} \text{Next},{\left(\lambda^{-}\right)}^{c}\left(x\cdot z\right)=-1-\lambda^{-}\left(x\cdot z\right)\geq -1-\text{max}\left\{\lambda^{-}\left(x\cdot \left(y^{*}z\right)\right),\lambda^{-}\left(y\right)\right\}\\ \;=\text{min}\left\{-1-\lambda^{-}\left(x\cdot \left(y^{*}z\right)\right),-1-\lambda^{-}\left(y\right)\right\}\\ \;=\text{min}\left\{{\left(\lambda^{-}\right)}^{c}\left(x\cdot \left(y^{*}z\right)\right),{\left(\lambda^{-}\right)}^{c}\left(y\right)\right\}.\\ \;\Rightarrow {\left(\lambda^{-}\right)}^{c}\left(x\cdot z\right)\geq \text{min}\left\{{\left(\lambda^{-}\right)}^{c}\left(x\cdot \left(y^{*}z\right)\right),{\left(\lambda^{-}\right)}^{c}\left(y\right)\right\}. \end{array}$$

$$\begin{array}{l} \text{Finally},{\left(\lambda^{-}\right)}^{c}\left(x^{*}z\right)=-1-\lambda^{-}\left(x^{*}z\right)\geq -1-\text{max}\left\{\lambda^{-}\left(x^{*}\left(y\cdot z\right)\right),\lambda^{-}\left(y\right)\right\}\\ \;=\text{min}\left\{-1-\lambda^{-}\left(x^{*}\left(y\cdot z\right)\right),-1-\lambda^{-}\left(y\right)\right\}\\ \;=\text{min}\left\{{\left(\lambda^{-}\right)}^{c}\left(x^{*}\left(y\cdot z\right)\right),{\left(\lambda^{-}\right)}^{c}\left(y\right)\right\}.\\ \;\Rightarrow {\left(\lambda^{-}\right)}^{c}\left(x^{*}z\right)\geq \text{min}\left\{{\left(\lambda^{-}\right)}^{c}\left(x^{*}\left(y\cdot z\right)\right),{\left(\lambda^{-}\right)}^{c}\left(y\right)\right\}. \end{array}$$

$$\begin{array}{l} {\left(\lambda^{-}\right)}^{c}\left(x\cdot z\right)=1-\lambda^{+}\left(x\cdot z\right)\leq 1-\text{min}\left\{\lambda^{+}\left(x\cdot \left(y^{*}z\right)\right),\lambda^{+}\left(y\right)\right\}\\ \;=\text{max}\left\{1-\lambda^{+}\left(x\cdot \left(y^{*}z\right)\right),1-\lambda^{+}\left(y\right)\right\}\\ \;=\text{max}\left\{{\left(\lambda^{-}\right)}^{c}\left(x\cdot \left(y^{*}z\right)\right),{\left(\lambda^{-}\right)}^{c}\left(y\right)\right\}.\\ \;\Rightarrow {\left(\lambda^{+}\right)}^{c}\left(x\cdot z\right)\leq \text{max}\left\{{\left(\lambda^{+}\right)}^{c}\left(x\cdot \left(y^{*}z\right)\right),{\left(\lambda^{+}\right)}^{c}\left(y\right)\right\}. \end{array}$$

$$\begin{array}{l} \text{And also},{\left(\lambda^{+}\right)}^{c}\left(x^{*}z\right)=1-\lambda^{+}\left(x^{*}z\right)\leq 1-\text{min}\left\{\lambda^{+}\left(x^{*}\left(y\cdot z\right)\right),\lambda^{+}\left(y\right)\right\}\\ \;=\text{max}\left\{1-\lambda^{+}\left(x^{*}\left(y\cdot z\right)\right),1-\lambda^{+}\left(y\right)\right\}\\ \;=\text{max}\left\{{\left(\lambda^{+}\right)}^{c}\left(x^{*}\left(y\cdot z\right)\right),{\left(\lambda^{+}\right)}^{c}\left(y\right)\right\}.\\ \;\Rightarrow {\left(\lambda^{+}\right)}^{c}\left(x^{*}z\right)\leq \text{max}\left\{{\left(\lambda^{+}\right)}^{c}\left(x^{*}\left(y\cdot z\right)\right),{\left(\lambda^{+}\right)}^{c}\left(y\right)\right\}. \end{array}$$

Therefore, *λ ^c^* is a bipolar fuzzy pseudo-UP ideal of *X.* □

**Table 1.** Bipolar fuzzy Pseudo-UP ideal.

·	0	a	b	c
0	0	a	b	c
a	0	0	b	c
b	0	a	0	c
c	0	a	0	0
*	0	a	b	c
0	0	a	b	c
a	0	0	b	c
b	0	a	0	c
c	0	a	b	0

**Table 2.** Bipolar fuzzy Pseudo-UP subalgebras is not bipolar fuzzy Pseudo-UP ideal.

·	0	1	2	3
0	0	1	2	3
1	0	0	1	3
2	0	0	0	3
3	0	0	0	0
*	0	1	2	3
0	0	1	2	3
1	0	0	1	3
2	0	0	0	3
3	0	0	2	0

**Table 3.** The union of bipolar fuzzy Pseudo-UP ideals is not bipolar fuzzy Pseudo-UP ideal.

·	0	1	2	3
0	0	1	2	3
1	0	0	3	3
2	0	1	0	0
3	0	1	3	0
*	0	1	2	3
0	0	1	2	3
1	0	0	3	3
2	0	1	0	0
3	0	2	3	0

## Homomorphism on bipolar fuzzy pseudo-UP ideal

In this section, we explore bipolar fuzzy pseudo-UP ideals in the context of homomorphism. We examine the homomorphic images and inverse images of bipolar fuzzy pseudo-UP ideals in pseudo-UP algebras, and present some key findings.

Theorem 4.1. Let *f*: *X*→ *Y* is an epimorphism of pseudo-UP algebras. If *λ* = ( *X*, *λ* ^+^, *λ* ^−^) is a bipolar fuzzy pseudo-UP ideal of *X* with sup-inf property, then the image f ( *λ*) is a bipolar pseudo-UP ideal of *Y.*

Proof. Assume that *λ* = ( *X*, *λ* ^+^, *λ* ^−^) is a bipolar fuzzy pseudo-UP ideal of *X* with sup-inf property. Let *x*, *y*, *z* ∈ *Y* with *a* ∈ *f* ^−1^( *x*), *b* ∈ *f* ^−1^( *y*) and *c* ∈ *f* ^−1^ ( *z*).

$$\lambda^{+}\left(a\right)=\underset{t\in f^{-1}\left(x\right)}{\sup}\lambda^{+}\left(t\right),\lambda^{+}\left(b\right)=\underset{t\in f^{-1}\left(y\right)}{\sup}\lambda^{+}\left(t\right)\;\text{and}\;\lambda^{+}\left(c\right)=\underset{t\in f^{-1}\left(z\right)}{\sup}\lambda^{+}\left(t\right)$$

$$\lambda^{-}\left(a\right)=\underset{t\in f^{-1}\left(x\right)}{\inf}\lambda^{-}\left(t\right),\lambda^{-}\left(b\right)=\underset{t\in f^{-1}\left(y\right)}{\inf}\lambda^{-}\left(t\right)\;\text{and}\;\lambda^{-}\left(c\right)=\underset{t\in f^{-1}\left(z\right)}{\inf}\lambda^{-}\left(t\right)$$

Then by Definition 2.7, we have

$$\left(f\right)\left(\lambda^{+}\right)\left(0\right)=\underset{t\in f^{-1}\left(0\right)}{\sup}\lambda^{+}\left(t\right)\geq \lambda^{+}\left(0\right)\geq \lambda^{+}\left(a\right)=\underset{t\in f^{-1}\left(x\right)}{\sup}\lambda^{+}\left(t\right)=\left(f\right)\left(\lambda^{+}\right)\left(x\right)$$

$$\left(f\right)\left(\lambda^{-}\right)\left(0\right)=\underset{t\in f^{-1}\left(0\right)}{\inf}\lambda^{-}\left(t\right)\geq \lambda^{-}\left(0\right)\geq \lambda^{-}\left(a\right)=\underset{t\in f^{-1}\left(x\right)}{\inf}\lambda^{-}\left(t\right)=\left(f\right)\left(\lambda^{-}\right)\left(x\right)$$

$$\begin{array}{l} \left(f\right)\left(\lambda^{+}\right)\left(x\cdot z\right)=\underset{t\in f^{-1}\left(x\cdot z\right)}{\sup}\lambda^{+}\left(t\right)=\lambda^{+}\left(a\cdot c\right)\\ \;\geq \text{min}\left\{\lambda^{+}\left(a\cdot \left(b^{*}c\right)\right),\lambda^{+}\left(b\right)\right\}\\ \;=\text{min}\left\{\underset{t\in f^{-1}\left(x\cdot \left(y^{*}z\right)\right)}{\sup}\lambda^{+}\left(t\right),\underset{t\in f^{-1}\left(y\right)}{\sup}\lambda^{+}\left(t\right)\right\}\\ \;=\text{min}\left\{\left(f\right)\left(\lambda^{+}\right)\left(x\cdot \left(y^{*}z\right)\right),\left(f\right)\left(\lambda^{+}\right)\left(y\right)\right\}.\\ \;\Rightarrow \left(f\right)\left(\lambda^{+}\right)\left(x\cdot z\right)\geq \text{min}\left\{\left(f\right)\left(\lambda^{+}\right)\left(x\cdot \left(y^{*}z\right)\right),\left(f\right)\left(\lambda^{+}\right)\left(y\right)\right\}. \end{array}$$

$$\begin{array}{l} \text{And also},\left(f\right)\left(\lambda^{+}\right)\left(x^{*}z\right)=\underset{t\in f^{-1}\left(x^{*}z\right)}{\sup}\lambda^{+}\left(t\right)=\lambda^{+}\left(a^{*}c\right)\\ \;\geq \text{min}\left\{\lambda^{+}\left(a^{*}\left(b\cdot c\right)\right),\lambda^{+}\left(b\right)\right\}\\ \;=\text{min}\left\{\underset{t\in f^{-1}\left(x^{*}\left(y\cdot z\right)\right)}{\sup}\lambda^{+}\left(t\right),\underset{t\in f^{-1}\left(y\right)}{\sup}\lambda^{+}\left(t\right)\right\}\\ \;=\text{min}\left\{\left(f\right)\left(\lambda^{+}\right)\left(x^{*}\left(y\cdot z\right)\right),\left(f\right)\left(\lambda^{+}\right)\left(y\right)\right\}.\\ \;\Rightarrow \left(f\right)\left(\lambda^{+}\right)\left(x^{*}z\right)\geq \text{min}\left\{\left(f\right)\left(\lambda^{+}\right)\left(x^{*}\left(y\cdot z\right)\right),\left(f\right)\left(\lambda^{+}\right)\left(y\right)\right\}. \end{array}$$

$$\begin{array}{l} \left(f\right)\left(\lambda^{-}\right)\left(x\cdot z\right)=\underset{t\in f^{-1}\left(x\cdot z\right)}{\inf}\lambda^{-}\left(t\right)=\lambda^{-}\left(a\cdot c\right)\\ \;\leq \text{max}\left\{\lambda^{-}\left(a\cdot \left(b^{*}c\right)\right),\lambda^{-}\left(b\right)\right\}\\ \;=\text{max}\left\{\underset{t\in f^{-1}\left(x\cdot \left(y^{*}z\right)\right)}{\inf}\lambda^{-}\left(t\right),\underset{t\in f^{-1}\left(y\right)}{\inf}\lambda^{-}\left(t\right)\right\}\\ \;=\text{max}\left\{\left(f\right)\left(\lambda^{-}\right)\left(x\cdot \left(y^{*}z\right)\right),\left(f\right)\left(\lambda^{-}\right)\left(y\right)\right\}.\\ \;\Rightarrow \left(f\right)\left(\lambda^{-}\right)\left(x\cdot z\right)\leq \text{max}\left\{\left(f\right)\left(\lambda^{-}\right)\left(x\cdot \left(y^{*}z\right)\right),\left(f\right)\left(\lambda^{-}\right)\left(y\right)\right\}. \end{array}$$

Finally, $\left(f\right)\left(\lambda^{-}\right)\left(x^{*}z\right)\leq \text{max}\left\{\left(f\right)\left(\lambda^{-}\right)\left(x^{*}\left(y\cdot z\right)\right),\left(f\right)\left(\lambda^{-}\right)\left(y\right)\right\}.$

Therefore, *f* ( *λ*) is a bipolar fuzzy pseudo-UP ideal of *Y.* □

Theorem 4.2. Let f be a homomorphism on pseudo-UP algebra *X* onto *Y* and *η* is a bipolar fuzzy pseudo-UP ideal of *Y.* Then *f* ^−1^ ( *η*) is a bipolar fuzzy pseudo-UP ideal of *X.*

Proof. Let f be a homomorphism of pseudo-UP algebra. Assume that *η* is a bipolar pseudo- UP ideal of *Y* and let *x* ∈ *X.* Then *f* ^−1^( *η* ^+^)(0) = *η* ^+^( *f*(0)) ≥ *η* ^+^( *f*( *x*)) = *f* ^−1^( *η* ^+^)( *x*) and *f* ^−1^( *η* ^−^)(0) = *η* ^−^( *f*(0)) ≤ *η* ^−^( *f*( *x*)) = *f* ^−1^( *η* ^−^)( *x*). Let *x*, *y*, *z* ∈ *X.* Then

$$\begin{array}{l} f^{-1}\left(\eta^{+}\right)\left(x\cdot z\right)=\eta^{+}\left(f\left(x\cdot z\right)\right)=\eta^{+}\left(f\left(x\right)\cdot f\left(z\right)\right)\\ \;\geq \text{min}\left\{\eta^{+}(f\left(x\right)\cdot \left(f\left(y\right)*f\left(z\right)\right),\eta^{+}\left(f\left(y\right)\right)\right\}\\ \;=\text{min}\left\{\eta^{+}(f\left(x\cdot \left(y^{*}z\right)\right),\eta^{+}\left(f\left(y\right)\right)\right\}\\ \;=\text{min}\left\{f^{-1}\left(\eta^{+}\left(x\cdot \left(y^{*}z\right)\right)\right),f^{-1}\left(\eta^{+}\left(y\right)\right)\right\}.\\ \;\Rightarrow f^{-1}\left(\eta^{+}\right)\left(x\cdot z\right)\geq \text{min}\left\{f^{-1}\left(\eta^{+}\left(x\cdot \left(y^{*}z\right)\right)\right),f^{-1}\left(\eta^{+}\left(y\right)\right)\right\}. \end{array}$$

$$\begin{array}{l} \text{And}\;f^{-1}\left(\eta^{+}\right)\left(x^{*}z\right)=\eta^{+}\left(f\left(x^{*}z\right)\right)=\eta^{+}\left(f\left(x\right)*f\left(z\right)\right)\\ \;\geq \text{min}\left\{\eta^{+}(f\left(x\right)*\left(f\left(y\right)\cdot f\left(z\right)\right),\eta^{+}\left(f\left(y\right)\right)\right\}\\ \;=\text{min}\left\{\eta^{+}(f\left(x^{*}\left(y\cdot z\right)\right),\eta^{+}\left(f\left(y\right)\right)\right\}\\ \;=\text{min}\left\{f^{-1}\left(\eta^{+}\left(x^{*}\left(y\cdot z\right)\right)\right),f^{-1}\left(\eta^{+}\left(y\right)\right)\right\}. \end{array}$$

$$\begin{array}{l} \text{And}f^{-1}\left(\eta^{-}\right)\left(x\cdot z\right)=\eta^{-}\left(f\left(x\cdot z\right)\right)=\eta^{-}\left(f\left(x\right)\cdot f\left(z\right)\right)\\ \;\leq \text{max}\left\{\eta^{-}(f\left(x\right)\cdot \left(f\left(y\right)*f\left(z\right)\right),\eta^{-}\left(f\left(y\right)\right)\right\}\\ \;=\text{max}\left\{\eta^{-}(f\left(x\cdot \left(y^{*}z\right)\right),\eta^{-}\left(f\left(y\right)\right)\right\}\\ \;=\text{max}\left\{f^{-1}\left(\eta^{-}\left(x\cdot \left(y^{*}z\right)\right)\right),f^{-1}\left(\eta^{-}\left(y\right)\right)\right\}. \end{array}$$

*Again*, $f^{-1}\left(\eta^{-}\right)\left(x^{*}z\right)\leq \text{max}\left\{f^{-1}\left(\eta^{-}\left(x^{*}\left(y\cdot z\right)\right)\right),f^{-1}\left(\eta^{-}\left(y\right)\right)\right\}.$

Hence, *f* ^−1^ ( *η*) is a bipolar fuzzy pseudo-UP ideal of *X.*

The next theorem shows that the converse of the above Theorem 4.2 is also true if *f* is an epimorphism of pseudo-UP algebra. □

Theorem 4.3. Let f: *X* → *Y* be an epimorphism of pseudo-UP algebras. Let *η* be a bipolar fuzzy subset of *Y.* If *f* ^−1^ ( *η*) is a bipolar fuzzy pseudo-UP ideal of *X*, then *η* is a bipolar fuzzy pseudo-UP ideal of *Y.*

Proof. Assume that *f* is an epimorphism of pseudo-UP algebra and *f* ^−1^ ( *η*) is a bipolar fuzzy pseudo- UP ideal of *X.* We need to show that *η* is a bipolar fuzzy pseudo-UP ideal of *Y.* Since f is an epimorphism of pseudo-UP algebra for any *x* ∈ *Y*, there exist *a* ∈ *X* such that *f* ( *a*) = *x.*

Then $\eta^{+}\left(0\right)=\eta^{+}\left(f\left(0\right)\right)=f^{-1}\left(\eta^{+}\right)\left(0)\right)\geq f^{-1}\left(\eta^{+}\right)\left(a\right)=\eta^{+}\left(f\left(a\right)\right)=\eta^{+}\left(x\right).$

$\eta^{-}\left(0\right)=\eta^{-}\left(f\left(0\right)\right)=f^{-1}\left(\eta^{-}\right)\left(0)\right)\leq f^{-1}\left(\eta^{-}\right)\left(a\right)=\eta^{-}\left(f\left(a\right)\right)=\eta^{-}\left(x\right).$

Let *x*, *y*, *z* ∈ *Y.* Then *f* ( *a*) = *x*, *f* ( *b*) = *y* and *f*( *c*) = *z*, for all *a*, *b*, *c* ∈ *X.* Thus

$$\begin{array}{l} \eta^{+}\left(x\cdot z\right)=\eta^{+}\left(f\left(x\right)\cdot f\left(z\right)\right)=\eta^{+}\left(f\left(a\cdot c\right)\right)\\ \;=f^{-1}\left(\eta^{+}\right)\left(a\cdot c\right)\\ \;\geq \text{min}\left\{f^{-1}\left(\eta^{+}\right)\left(a\cdot \left(b^{*}c\right)\right),f^{-1}\left(\eta^{+}\right)\left(b\right)\right\}\\ \;=\text{min}\left\{\eta^{+}(f\left(a\cdot \left(b^{*}c\right)\right),\eta^{+}\left(f\left(b\right)\right)\right\}\\ \;=\text{min}\left\{\eta^{+}\left(f\left(a\right)\cdot \left(f\left(b\right)*f\left(c\right)\right)\right),\eta^{+}\left(f\left(b\right)\right)\right\}\\ \;=\text{min}\left\{\eta^{+}\left(x\cdot \left(y^{*}z\right)\right),\eta^{+}\left(y\right)\right\}. \end{array}$$

$$\begin{array}{l} \eta^{+}\left(x^{*}z\right)=\eta^{+}\left(f\left(x\right)\cdot f\left(z\right)\right)=\eta^{+}\left(f\left(a^{*}c\right)\right)\\ \;=f^{-1}\left(\eta^{+}\right)\left(a^{*}c\right)\\ \;\geq \text{min}\left\{f^{-1}\left(\eta^{+}\right)\left(a^{*}\left(b\cdot c\right)\right),f^{-1}\left(\eta^{+}\right)\left(b\right)\right\}\\ \;=\text{min}\left\{\eta^{+}(f\left(a^{*}\left(b\cdot c\right)\right),\eta^{+}\left(f\left(b\right)\right)\right\}\\ \;=\text{min}\left\{\eta^{+}\left(f\left(a\right)*\left(f\left(b\right)\cdot f\left(c\right)\right)\right),\eta^{+}\left(f\left(b\right)\right)\right\}\\ \;=\text{min}\left\{\eta^{+}\left(x^{*}\left(y\cdot z\right)\right),\eta^{+}\left(y\right)\right\}.\\ \;\text{And} \end{array}$$

$$\begin{array}{l} \eta^{-}\left(x\cdot z\right)=\eta^{-}\left(f\left(x\right)\cdot f\left(z\right)\right)=\eta^{-}\left(f\left(a\cdot c\right)\right)\\ \;=f^{-1}\left(\eta^{-}\right)\left(a\cdot c\right)\\ \;\leq \text{max}\left\{f^{-1}\left(\eta^{-}\right)\left(a\cdot \left(b^{*}c\right)\right),f^{-1}\left(\eta^{-}\right)\left(b\right)\right\}\\ \;=\text{max}\left\{\eta^{-}(f\left(a\cdot \left(b^{*}c\right)\right),\eta^{-}\left(f\left(b\right)\right)\right\}\\ \;=\text{max}\left\{\eta^{-}\left(f\left(a\right)\cdot \left(f\left(b\right)*f\left(c\right)\right)\right),\eta^{-}\left(f\left(b\right)\right)\right\}\\ \;=\text{max}\left\{\eta^{-}\left(x\cdot \left(y^{*}z\right)\right),\eta^{-}\left(y\right)\right\}. \end{array}$$

Similarly, $\eta^{-}\left(x\cdot z\right)\leq \text{max}\left\{\eta^{-}\left(x\cdot \left(y^{*}z\right)\right),\eta^{-}\left(y\right)\right\}$ .

Therefore, *η* is a bipolar fuzzy pseudo-UP ideal of *Y.* □

## Cartesian products of bipolar fuzzy pseudo-UP ideal

In this section, we discuss the Cartesian product of two bipolar fuzzy pseudo-UP ideal is also again a bipolar fuzzy pseudo-UP ideal and some other results are also investigated.

Theorem 5.1. Let *λ* = ( *X*, *λ* ^+^, *λ* ^−^) and *σ* = ( *Y*, *σ* ^+^, *σ* ^−^) be any two bipolar fuzzy pseudo-UP ideals of *X* and *Y* respectively. Then *λ* × *σ* is a bipolar fuzzy pseudo-UP ideal of *X* × *Y.*

Proof. Assume that *λ* and *σ* be any two bipolar fuzzy pseudo-UP ideals of *X* and *Y* respectively.

Take ( *x*, *y*) ∈ *X* × *Y.* Then

$$\begin{array}{l} \lambda^{+}\times \sigma^{+}\left(0,0\right)=\text{min}\left\{\lambda^{+}\left(0\right),\sigma^{+}\left(0\right)\right\}\\ \;\geq \text{min}\left\{\lambda^{+}\left(x\right),\sigma^{+}\left(y\right)\right\}\\ \;=\lambda^{+}\times \sigma^{+}\left(x,y\right). \end{array}$$

$$\begin{array}{l} \text{And}\;\lambda^{-}\times \sigma^{-}\left(0,0\right)=\text{max}\left\{\lambda^{-}\left(0\right),\sigma^{-}\left(0\right)\right\}\\ \;\leq \text{max}\left\{\lambda^{-}\left(x\right),\sigma^{-}\left(y\right)\right\}\\ \;=\lambda^{-}\times \sigma^{-}\left(x,y\right). \end{array}$$

Let ( *x*_1_, *x*_2_), ( *y*_1_, *y*_2_), ( *z*_1_, *z*_2_) ∈ *X* × *Y.* Put *x* = ( *x*_1_, *x*_2_), *y* = ( *y*_1_, *y*_2_), *z* = ( *z*_1_, *z*_2_). Then,

$$\begin{array}{l} \lambda^{+}\times \sigma^{+}\left(x\cdot z\right)=\lambda^{+}\times \sigma^{+}\left(\left(x_{1},x_{2}\right)\cdot \left(z_{1},z_{2}\right)\right)\\ \;=\lambda^{+}\times \sigma^{+}\left(x_{1}\cdot z_{1},x_{2}\cdot z_{2}\right)\\ \;=\text{min}\left\{\lambda^{+}\left(x_{1}\cdot z_{1}\right),\sigma^{+}\left(x_{2},z_{2}\right)\right\}\\ \;\geq \text{min}\left\{\text{min}\left\{\lambda^{+}\left(x_{1}\cdot \left(y_{1}*z_{1}\right)\right),\lambda^{+}\left(y_{1}\right)\right\},\text{min}\left\{\sigma^{+}\left(x_{2}\cdot \left(y_{2}*z_{2}\right)\right),\sigma^{+}\left(y_{2}\right)\right\}\right\}\\ \;=\text{min}\left\{\text{min}\left\{\lambda^{+}\left(x_{1}\cdot \left(y_{1}*z_{1}\right)\right),\sigma^{+}\left(x_{2}\cdot \left(y_{2}*z_{2}\right)\right)\right\},\text{min}\left\{\lambda^{+}\left(y_{1}\right),\sigma^{+}\left(y_{2}\right)\right\}\right\}\\ \;=\text{min}\left\{\lambda^{+}\times \sigma^{+}\left((x_{1}\cdot \left(y_{1}*z_{1}\right)\right),(x_{2}\cdot \left(y_{2}*z_{2}\right)),\lambda^{+}\times \sigma^{+}\left(y_{1},y_{2}\right)\right\}\\ \;=\text{min}\left\{\lambda^{+}\times \sigma^{+}\left((x_{1},x_{2}\right)\cdot \left(\left(y_{1},y_{2}\right)*\left((z_{1},z_{2})\right)\right),\lambda^{+}\times \sigma^{+}\left(y_{1},y_{2}\right)\right\}\\ \;=\text{min}\left\{\lambda^{+}\times \sigma^{+}\left(x\cdot \left(y*z\right)\right),\lambda^{+}\times \sigma^{+}\left(y\right)\right\}. \end{array}$$

And, $\lambda^{+}\times \sigma^{+}\left(x^{*}z\right)\geq \text{min}\left\{\lambda^{+}\times \sigma^{+}\left(x\cdot \left(y^{*}z\right)\right),\lambda^{+}\times \sigma^{+}\left(y\right)\right\}$ .

And,

$$\begin{array}{l} \lambda^{-}\times \sigma^{-}\left(x\cdot z\right)=\lambda^{-}\times \sigma^{-}\left(\left(x_{1},x_{2}\right)\cdot \left(z_{1},z_{2}\right)\right)\\ \;=\lambda^{-}\times \sigma^{-}\left(x_{1}\cdot z_{1},x_{2}\cdot z_{2}\right)\\ \;=\text{max}\left\{\lambda^{-}\left(x_{1}\cdot z_{1}\right),\sigma^{-}\left(x_{2},z_{2}\right)\right\}\\ \;\leq \text{max}\left\{\text{max}\left\{\lambda^{-}\left(x_{1}\cdot \left(y_{1}*z_{1}\right)\right),\lambda^{-}\left(y_{1}\right)\right\},\text{max}\left\{\sigma^{-}\left(x_{2}\cdot \left(y_{2}*z_{2}\right)\right),\sigma^{-}\left(y_{2}\right)\right\}\right\}\\ \;=\text{max}\left\{\text{max}\left\{\lambda^{-}\left(x_{1}\cdot \left(y_{1}*z_{1}\right)\right),\sigma^{-}\left(x_{2}\cdot \left(y_{2}*z_{2}\right)\right)\right\},\text{max}\left\{\lambda^{-}\left(y_{1}\right),\sigma^{-}\left(y_{2}\right)\right\}\right\}\\ \;=\text{max}\left\{\lambda^{-}\times \sigma^{-}\left((x_{1}\cdot \left(y_{1}*z_{1}\right)\right),(x_{2}\cdot \left(y_{2}*z_{2}\right)),\lambda^{-}\times \sigma^{-}\left(y_{1},y_{2}\right)\right\}\\ \;=\text{max}\left\{\lambda^{-}\times \sigma^{-}\left((x_{1},x_{2}\right)\cdot \left(\left(y_{1},y_{2}\right)*\left((z_{1},z_{2})\right)\right),\lambda^{-}\times \sigma^{-}\left(y_{1},y_{2}\right)\right\}\\ \;=\text{max}\left\{\lambda^{-}\times \sigma^{-}\left(x\cdot \left(y*z\right)\right),\lambda^{-}\times \sigma^{-}\left(y\right)\right\}. \end{array}$$

Additionally, $\lambda^{-}\times \sigma^{-}\left(x^{*}z\right)\leq \text{max}\left\{\lambda^{-}\times \sigma^{-}\left(x\cdot \left(y^{*}z\right)\right),\lambda^{-}\times \sigma^{-}\left(y\right)\right\}.$

Hence, *λ* × *σ* is a bipolar fuzzy pseudo-UP ideal of *X* × *Y.* □

Lemma 5.2. Let *λ* and *σ* be two bipolar fuzzy subsets of *X* and *Y.* If *λ* × *σ* is a bipolar fuzzy pseudo-UP ideal of *X* × *Y.* Then the followings are true.

1) *λ* ^+^(0) ≥ *σ* ^+^( *y*) and *σ* ^+^(0) ≥ *λ* ^+^( *x*),
2) *λ* ^−^ (0) ≤ *σ* ^−^( *y*) and *σ* ^−^ (0) ≤ *λ* ^−^( *x*).

Proof. 1) Assume *σ* ^+^( *y*) ≥ *λ* ^+^ (0) and *λ* ^+^( *x*) ≥ *σ* ^+^ (0), ∀ *x* ∈ *X*, *y* ∈ *Y*

$$\begin{array}{l} \text{Then},\lambda^{+}\times \sigma^{+}\left(x,y\right)=\text{min}\left\{\lambda^{+}\left(x\right),\sigma^{+}\left(y\right)\right\}\\ \geq \text{min}\left\{\lambda^{+}\left(0\right),\sigma^{+}\left(0\right)\right\}\\ =\lambda^{+}\times \sigma^{+}\left(0,0\right)\;\text{which is}\;a\;\text{contradiction}. \end{array}$$

2) Similarly, $\lambda^{-}\left(x\right)\leq \sigma^{-}\left(0\right)\;\text{and}\;\sigma^{-}\left(y\right)\leq \lambda^{-}\left(y\right),\forall x\in X,y\in Y$

$$\begin{array}{l} \text{Then},\lambda^{-}\times \sigma^{-}\left(x,y\right)=\text{max}\left\{\lambda^{-}\left(x\right),\sigma^{-}\left(y\right)\right\}\\ \leq \text{max}\left\{\lambda^{-}\left(0\right),\sigma^{-}\left(0\right)\right\}\\ =\lambda^{-}\times \sigma^{-}\left(0,0\right)\;\text{which is}\;a\;\text{contradiction}. \end{array}$$

Thus proving the result. □

Theorem 5.3. If *λ* × *σ* is a bipolar fuzzy pseudo-UP ideal of *X* × *Y* for any two bipolar fuzzy subsets *λ* and *σ* of *X* and *Y* respectively, then either *λ* is a bipolar fuzzy pseudo-UP ideal of *X* or *σ* is a bipolar fuzzy pseudo-UP ideal of *Y.*

Proof. Let *λ* and *σ* be any two bipolar fuzzy subsets of pseudo-UP algebra *X* and *Y* respectively such that *λ* × *σ* is a bipolar fuzzy pseudo-UP ideal of *X* × *Y.* Then *λ* ^+^ × *σ* ^+^ (0, 0) ≥ *λ* ^+^ × *σ* ^+^( *x*, *y*). Now by Lemma 5.2, let *λ* ^+^ (0) ≥ *λ* ^+^( *x*) or *σ* ^+^ (0) ≥ *σ* ^+^( *y*) and *λ* ^−^ (0) ≤ *λ* ^−^( *x*) or *σ* ^−^ (0) ≤ *σ* ^−^( *y*).

Let ( *x*_1_, *x*_2_), ( *y*_1_, *y*_2_), ( *z*_1_, *z*_2_) ∈ *X* × *Y.* Put *x* = ( *x*_1_, *x*_2_), *y* = ( *y*_1_, *y*_2_), *z* = ( *z*_1_, *z*_2_).

$$\begin{array}{l} \text{Then}\;\lambda^{+}\times \sigma^{+}\left(x\cdot z\right)\geq \text{min}\left\{\lambda^{+}\times \sigma^{+}\left(x\cdot \left(y^{*}z\right)\right),\lambda^{+}\times \sigma^{+}\left(y\right)\right\}\\ \;=\text{min}\left\{\lambda^{+}\times \sigma^{+}\left((x_{1},x_{2}\right)\cdot \left(\left(y_{1},y_{2}\right)*\left(z_{1},z_{2}\right)\right),\lambda^{+}\times \sigma^{+}\left(y_{1},y_{2}\right)\right\}\\ \;=\text{min}\left\{\lambda^{+}\times \sigma^{+}((x_{1}\cdot \left({y_{1}}^{*}z_{1}\right),\left(x_{2}\cdot \left({y_{2}}^{*}z_{2}\right)\right)\sigma^{+}\times \sigma^{+}\left(y_{1},y_{2}\right)\right\}\\ \;=\text{min}\left\{\text{min}\left\{\lambda^{+}\left({x_{1}}^{*}\left({y_{1}}^{*}z_{1}\right)\right),\sigma^{+}\left(x_{2}\cdot \left({y_{2}}^{*}z_{2}\right)\right)\right\},\text{min}\left\{\lambda^{+}\left(y_{1}\right),\sigma^{+}\left(y_{2}\right)\right\}\right\} \end{array}$$

$$\begin{array}{l} \lambda^{+}\times \sigma^{+}\left(x_{1}\cdot z_{1},x_{2}\cdot z_{2}\right)\geq \text{min}\left\{\text{min}\left\{\lambda^{+}\left({x_{1}}^{*}\left({y_{1}}^{*}z_{1}\right)\right),\sigma^{+}\left(x_{2}\cdot \left({y_{2}}^{*}z_{2}\right)\right)\right\},\text{min}\left\{\lambda^{+}\left(y_{1}\right),\sigma^{+}\left(y_{2}\right)\right\}\right\}\\ \;=\text{min}\left\{\text{min}\left\{\lambda^{+}({x_{1}}^{*}\left({y_{1}}^{*}z_{1}\right),\lambda^{+}\left(y_{1}\right)\right\},\text{min}\left\{\sigma^{+}\left(x_{2}\cdot \left({y_{2}}^{*}z_{2}\right)\right),\sigma^{+}\left(y_{2}\right)\right\}\right\}. \end{array}$$

⇒ either *λ* ^+^( *x*_1_ · *z*_1_) ≥ min { *λ* ^+^( *x*_1_ · ( *y*_1_ * *z*_1_)), *λ* ^+^ ( *y*_1_)} or *σ* ^+^( *x*_2_ · *z*_2_) ≥ min { *σ* ^+^( *x*_2_ · ( *y*_2_ * *z*_2_)), *σ* ^+^ ( *y*_2_)}. And either *λ* ^+^( *x*_1_* *z*_1_) ≥ min { *λ* ^+^( *x*_1_*( *y*_1_· *z*_1_)), *λ* ^+^ ( *y*_1_)} or *σ* ^+^( *x*_2_* *z*_2_) ≥ min { *σ* ^+^( *x*_2_*( *y*_2_· *z*_2_)), *σ* ^+^ ( *y*_2_)}. Similarly we can show that either *λ* ^−^( *x*_1_ · *z*_1_) ≤ max { *λ* ^−^( *x*_1_ · ( *y*_1_ * *z*_1_)), *λ* ^−^( *y*_1_)} or *σ* ^−^( *x*_2_ · *z*_2_) ≤ max { *σ* ^−^( *x*_2_ · ( *y*_2_ * *z*_2_)), *σ* ^−^( *y*_2_)} and *λ* ^−^( *x*_1_ * *z*_1_) ≤ max { *λ* ^−^( *x*_1_ * ( *y*_1_ · *z*_1_)), *λ* ^−^( *y*_1_)} or *σ* ^−^( *x*_2_ * *z*_2_) ≤ max { *σ* ^−^( *x*_2_ * ( *y*_2_ · *z*_2_)), *σ* ^−^( *y*_2_)}. Hence, either *λ* is a bipolar fuzzy pseudo-UP ideal of *X* or *σ* is a bipolar fuzzy pseudo-UP ideal of *Y.* □

Theorem 5.4. Let *λ* and *σ* be any bipolar fuzzy subsets of *X* and *Y* respectively. Then *λ* × *σ* is a bipolar fuzzy pseudo-UP ideal of *X* × *Y* if and only If *λ* ^+^ × *σ* ^+^ and ( *λ* ^−^ × *σ* ^−^) *^c^* are fuzzy pseudo-UP ideals of *X* × *Y.*

Proof. Let *λ* and *σ* be any two bipolar fuzzy subsets of *X* and *Y* respectively such that *λ* × *σ* is a bipolar fuzzy pseudo-UP ideal of *X* × *Y.* Now we need to show that *λ* ^+^ × *σ* ^+^ and ( *λ* ^−^ × *σ* ^−^) *^c^* are fuzzy pseudo-UP ideals of *X* × *Y.* Clearly, *λ* ^+^ × *σ* ^+^ is a fuzzy pseudo-UP ideal of *X* × *Y.* So it remains to show that ( *λ* ^−^ × *σ* ^−^) *^c^* is a fuzzy pseudo-UP ideal of *X* × *Y.*

Now, for every ( *x*_1_, *x*_2_), ( *y*_1_, *y*_2_), ( *z*_1_, *z*_2_) ∈ *X* × *Y.*

$${\left(\lambda^{-}\times \sigma^{-}\right)}^{c}\left(0,0\right)=-1-\lambda^{-}\times \sigma^{-}\left(0,0\right)\geq -1-\lambda^{-}\times \sigma^{-}\left(x_{1},x_{2}\right)={\left(\lambda^{-}\times \sigma^{-}\right)}^{c}\left(x_{1},x_{2}\right).$$

$$\begin{array}{l} {\left(\lambda^{-}\times \sigma^{-}\right)}^{c}\left((x_{1},x_{2}\right)\cdot \left((z_{1},z_{2})\right)=-1-\lambda^{-}\times \sigma^{-}\left((x_{1},x_{2}\right)\cdot \left((z_{1},z_{2})\right)\\ \;\geq -1-\text{max}\left\{\lambda^{-}\times \sigma^{-}\left(\left(x_{1},x_{2}\right)\cdot \left(\left(y_{1},y_{2}\right)*\left(z_{1},z_{2}\right)\right)\right),\lambda^{-}\times \sigma^{-}\left(y_{1},y_{2}\right)\right\}\\ \;=\text{min}\left\{-1-\lambda^{-}\times \sigma^{-}\left(\left(x_{1},x_{2}\right)\cdot \left(\left(y_{1},y_{2}\right)*\left(z_{1},z_{2}\right)\right)\right),-1-\lambda^{-}\times \sigma^{-}\left(y_{1},y_{2}\right)\right\}\\ \;=\text{min}\left\{{\left(\lambda^{-}\times \sigma^{-}\right)}^{c}\left(\left(x_{1},x_{2}\right)\cdot \left(\left(y_{1},y_{2}\right)*\left(z_{1},z_{2}\right)\right)\right),{\left(\lambda^{-}\times \sigma^{-}\right)}^{c}\left(y_{1},y_{2}\right)\right\}. \end{array}$$

$$\begin{array}{l} {\left(\lambda^{-}\times \sigma^{-}\right)}^{c}\left((x_{1},x_{2}\right)*\left((z_{1},z_{2})\right)=-1-\lambda^{-}\times \sigma^{-}\left((x_{1},x_{2}\right)*\left((z_{1},z_{2})\right)\\ \;\geq -1-\text{max}\left\{\lambda^{-}\times \sigma^{-}\left(\left(x_{1},x_{2}\right)*\left(\left(y_{1},y_{2}\right)\cdot \left(z_{1},z_{2}\right)\right)\right),\lambda^{-}\times \sigma^{-}\left(y_{1},y_{2}\right)\right\}\\ \;=\text{min}\left\{-1-\lambda^{-}\times \sigma^{-}\left(\left(x_{1},x_{2}\right)*\left(\left(y_{1},y_{2}\right)\cdot \left(z_{1},z_{2}\right)\right)\right),-1-\lambda^{-}\times \sigma^{-}\left(y_{1},y_{2}\right)\right\}\\ \;=\text{min}\left\{{\left(\lambda^{-}\times \sigma^{-}\right)}^{c}\left(\left(x_{1},x_{2}\right)*\left(\left(y_{1},y_{2}\right)\cdot \left(z_{1},z_{2}\right)\right)\right),{\left(\lambda^{-}\times \sigma^{-}\right)}^{c}\left(y_{1},y_{2}\right)\right\}. \end{array}$$

Hence, ( *λ* ^−^ × *σ* ^−^) *^c^* is a fuzzy pseudo-UP ideal of *X* × *Y.*

Conversely, assume that *λ* ^+^ × *σ* ^+^ and ( *λ* ^−^ × *σ* ^−^) *^c^* are fuzzy pseudo-UP ideals of *X* × *Y.* We need to show that *λ* × *σ* is a bipolar fuzzy pseudo-UP ideal of *X* × *Y.* Let ( *x*_1_, *x*_2_), ( *y*_1_, *y*_2_), ( *z*_1_, *z*_2_) ∈ *X* × *Y.* Then $\lambda^{+}\times \sigma^{+}\left(0,0\right)\geq \lambda^{+}\times \sigma^{+}\left(x_{1},x_{2}\right)\;\text{and}\;\lambda^{-}\times \sigma^{-}\left(0,0\right)\leq \lambda^{-}\times \sigma^{-}\left(x_{1},x_{2}\right)$ .

Now, we have ${\left(\lambda^{-}\times \sigma^{-}\right)}^{c}\left(0,0\right)\geq {\left(\lambda^{-}\times \sigma^{-}\right)}^{c}\left(x_{1},x_{2}\right)\Rightarrow -1-\lambda^{-}\times \sigma^{-}\left(0,0\right)\geq -1-\lambda^{-}\times \sigma^{-}\left(x_{1},x_{2}\right)\Rightarrow \left(\lambda^{-}\times \sigma^{-}\right)\left(0,0\right)\leq \left(\lambda^{-}\times \sigma^{-}\right)\left(x_{1},x_{2}\right)$

$\lambda^{+}\times \sigma^{+}\left(\left(x_{1},x_{2}\right)\cdot \left(z_{1},z_{2}\right)\right)\geq \text{min}\left\{\lambda^{+}\times \sigma^{+}\left(\left(x_{1},x_{2}\right)\cdot \left(\left(y_{1},y_{2}\right)*\left(z_{1},z_{2}\right)\right)\right),\lambda^{+}\times \sigma^{+}\left(y_{1},y_{2}\right)\right\}.$

And, $\lambda^{+}\times \sigma^{+}\left(\left(x_{1},x_{2}\right)*\left(z_{1},z_{2}\right)\right)\geq \text{min}\left\{\lambda^{+}\times \sigma^{+}\left(\left(x_{1},x_{2}\right)*\left(\left(y_{1},y_{2}\right)\cdot \left(z_{1},z_{2}\right)\right)\right),\lambda^{+}\times \sigma^{+}\left(y_{1},y_{2}\right)\right\}$ .

$$\begin{array}{l} -1-\lambda^{-}\times \sigma^{-}\left(\left(x_{1},x_{2}\right)\cdot \left(z_{1},z_{2}\right)\right)={\left(\lambda^{-}\times \sigma^{-}\right)}^{c}\left(\left(x_{1},x_{2}\right)\cdot \left(z_{1},z_{2}\right)\right)\\ \;\geq \text{min}\left\{{\left(\lambda^{-}\times \sigma^{-}\right)}^{c}\left(\left(x_{1},x_{2}\right)\cdot \left(\left(y_{1},y_{2}\right)*\left(z_{1},z_{2}\right)\right)\right),{\left(\lambda^{-}\times \sigma^{-}\right)}^{c}\left(y_{1},y_{2}\right)\right\}\\ \;=\text{min}\left\{-1-\lambda^{-}\times \sigma^{-}\left(\left(x_{1},x_{2}\right)\cdot \left(\left(y_{1},y_{2}\right)*\left(z_{1},z_{2}\right)\right)\right),-1-\lambda^{-}\times \sigma^{-}\left(y_{1},y_{2}\right)\right\}\\ \;=-1-\text{max}\left\{\lambda^{-}\times \sigma^{-}\left(\left(x_{1},x_{2}\right)\cdot \left(\left(y_{1},y_{2}\right)*\left(z_{1},z_{2}\right)\right)\right),\lambda^{-}\times \sigma^{-}\left(y_{1},y_{2}\right)\right\} \end{array}$$

$$\Rightarrow \lambda^{-}\times \sigma^{-}\left(\left(x_{1},x_{2}\right)\cdot \left(z_{1},z_{2}\right)\right)\leq \text{max}\left\{\lambda^{-}\times \sigma^{-}\left(\left(x_{1},x_{2}\right)\cdot \left(\left(y_{1},y_{2}\right)*\left(z_{1},z_{2}\right)\right)\right),\lambda^{-}\times \sigma^{-}\left(y_{1},y_{2}\right)\right\}.$$

Similarly, $\lambda^{-}\times \sigma^{-}\left(\left(x_{1},x_{2}\right)*\left(z_{1},z_{2}\right)\right)\leq \text{max}\left\{\lambda^{-}\times \sigma^{-}\left(\left(x_{1},x_{2}\right)*\left(\left(y_{1},y_{2}\right)\cdot \left(z_{1},z_{2}\right)\right)\right),\lambda^{-}\times \sigma^{-}\left(y_{1},y_{2}\right)\right\}$ .

Hence, *λ* × *σ* is a bipolar fuzzy pseudo-UP ideal of *X* × *Y.* □

Theorem 5.5. Let *λ* and *σ* be any two bipolar fuzzy subsets of *X* and *Y*, respectively. Then *λ* × *σ* is a bipolar fuzzy pseudo-UP ideal of *X* × *Y* if and only if □ ( *λ* × *σ*) = ( *X* × *Y*, *λ* ^+^ × *σ* ^+^, ( *λ* ^+^ × *σ* ^+^) *^c^*) and ♦ ( *λ* × *σ*) = ( *X* × *Y*, ( *λ* ^−^ × *σ* ^−^) *^c^*, ( *λ* ^−^ × *σ* ^−^) are bipolar fuzzy pseudo-UP ideals of *X* × *Y.*

Proof. Assume that *λ* × *σ* is a bipolar fuzzy pseudo-UP ideal of *X* × *Y.* Now we need to show that ( *λ*× *σ*) and ♦ ( *λ*× *σ*) are bipolar fuzzy pseudo-UP ideal of *X*× *Y.* Then for any ( *x*_1_, *x*_2_), ( *y*_1_, *y*_2_), ( *z*_1_, *z*_2_) ∈ *X* × *Y*, we have *λ* ^+^ × *σ* ^+^(0, 0) ≥ *λ* ^+^ × *σ* ^+^( *x*_1_, *x*_2_), *λ* ^+^ × *σ* ^+^(( *x*_1_, *x*_2_) · (( *z*_1_, *z*_2_)) ≥ min { *λ* ^+^ × *σ* ^+^(( *x*_1_, *x*_2_) · (( *y*_1_, *y*_2_)*( *z*_1_, *z*_2_))), *λ* ^+^× *σ* ^+^( *y*_1_, *y*_2_)} and *λ* ^+^× *σ* ^+^(( *x*_1_, *x*_2_)*(( *z*_1_, *z*_2_)) ≥ min { *λ* ^+^× *σ* ^+^(( *x*_1_, *x*_2_)*(( *y*_1_, *y*_2_)· ( *z*_1_, *z*_2_))), *λ* ^+^ × *σ* ^+^( *y*_1_, *y*_2_)}.

Next, let ( *x*_1_, *x*_2_), ( *y*_1_, *y*_2_), ( *z*_1_, *z*_2_) ∈ *X* × *Y.* we have ( *λ* ^+^ × *σ* ^+^) *^c^* (0, 0) = 1 − *λ* ^+^ × *σ* ^+^ (0, 0) ≤1 − *λ* ^+^ × *σ* ^+^( *x*_1_, *x*_2_) = ( *λ* ^+^ × *σ* ^+^) *^c^* ( *x*_1_, *x*_2_).

$$\begin{array}{l} {\left(\lambda^{+}\times \sigma^{+}\right)}^{c}\left(\left(x_{1},x_{2}\right)\cdot \left(z_{1},z_{2}\right)\right)=1-\lambda^{+}\times \sigma^{+}\left(\left(x_{1},x_{2}\right)\cdot \left(z_{1},z_{2}\right)\right)\\ \;\leq 1-\text{min}\left\{\lambda^{+}\times \sigma^{+}\left(\left(x_{1},x_{2}\right)\cdot \left(\left(y_{1},y_{2}\right)*\left(x_{1},z_{2}\right)\right)\right),\lambda^{+}\times \sigma^{+}\left(y_{1},y_{2}\right)\right\}\\ \;=\text{max}\left\{1-\lambda^{+}\times \sigma^{+}\left(\left(x_{1},x_{2}\right)\cdot \left(\left(y_{1},y_{2}\right)*\left(x_{1},z_{2}\right)\right)\right),1-\lambda^{+}\times \sigma^{+}\left(y_{1},y_{2}\right)\right\}\\ \;=\text{max}\left\{{\left(\lambda^{+}\times \sigma^{+}\right)}^{c}\left(\left(x_{1},x_{2}\right)\cdot \left(\left(y_{1},y_{2}\right)*\left(x_{1},z_{2}\right)\right)\right),{\left(\lambda^{+}\times \sigma^{+}\right)}^{c}\left(y_{1},y_{2}\right)\right\}. \end{array}$$

$$\begin{array}{l} {\left(\lambda^{+}\times \sigma^{+}\right)}^{c}\left(\left(x_{1},x_{2}\right)*\left(z_{1},z_{2}\right)\right)=1-\lambda^{+}\times \sigma^{+}\left(\left(x_{1},x_{2}\right)*\left(z_{1},z_{2}\right)\right)\\ \;\leq 1-\text{min}\left\{\lambda^{+}\times \sigma^{+}\left(\left(x_{1},x_{2}\right)*\left(\left(y_{1},y_{2}\right)\cdot \left(x_{1},z_{2}\right)\right)\right),\lambda^{+}\times \sigma^{+}\left(y_{1},y_{2}\right)\right\}\\ \;=\text{max}\left\{1-\lambda^{+}\times \sigma^{+}\left(\left(x_{1},x_{2}\right)*\left(\left(y_{1},y_{2}\right)\cdot \left(x_{1},z_{2}\right)\right)\right),1-\lambda^{+}\times \sigma^{+}\left(y_{1},y_{2}\right)\right\}\\ \;=\text{max}\left\{{\left(\lambda^{+}\times \sigma^{+}\right)}^{c}\left(\left(x_{1},x_{2}\right)*\left(\left(y_{1},y_{2}\right)\cdot \left(x_{1},z_{2}\right)\right)\right),{\left(\lambda^{+}\times \sigma^{+}\right)}^{c}\left(y_{1},y_{2}\right)\right\}. \end{array}$$

Hence, ( *λ* × *σ*) is a bipolar fuzzy pseudo-UP ideal of *X* × *Y.*

And, ${\left(\lambda^{-}\times \sigma^{-}\right)}^{c}\left(0,0\right)=-1-\lambda^{-}\times \sigma^{-}\left(0,0\right)\geq -1-\lambda^{-}\times \sigma^{-}\left(x_{1},x_{2}\right)={\left(\lambda^{-}\times \sigma^{-}\right)}^{c}\left(x_{1},x_{2}\right)$ .

$$\begin{array}{l} {\left(\lambda^{-}\times \sigma^{-}\right)}^{c}\left(\left(x_{1},x_{2}\right)\cdot \left(z_{1},z_{2}\right)\right)=-1-\lambda^{-}\times \sigma^{-}\left(\left(x_{1},x_{2}\right)\cdot \left(z_{1},z_{2}\right)\right)\\ \;\geq -1-\text{max}\left\{\lambda^{-}\times \sigma^{-}\left(\left(x_{1},x_{2}\right)\cdot \left(\left(y_{1},y_{2}\right)*\left(z_{1},z_{2}\right)\right)\right),\lambda^{-}\times \sigma^{-}\left(y_{1},y_{2}\right)\right\}\\ \;=\text{min}\left\{-1-\lambda^{-}\times \sigma^{-}\left(\left(x_{1},x_{2}\right)\cdot \left(\left(y_{1},y_{2}\right)*\left(z_{1},z_{2}\right)\right)\right),-1-\lambda^{-}\times \sigma^{-}\left(y_{1},y_{2}\right)\right\}\\ \;=\text{min}\left\{{\left(\lambda^{-}\times \sigma^{-}\right)}^{c}\left(\left(x_{1},x_{2}\right)\cdot \left(\left(y_{1},y_{2}\right)*\left(z_{1},z_{2}\right)\right)\right),{\left(\lambda^{-}\times \sigma^{-}\right)}^{c}\left(y_{1},y_{2}\right)\right\}. \end{array}$$

$$\begin{array}{l} {\left(\lambda^{-}\times \sigma^{-}\right)}^{c}\left(\left(x_{1},x_{2}\right)*\left(z_{1},z_{2}\right)\right)=-1-\lambda^{-}\times \sigma^{-}\left(\left(x_{1},x_{2}\right)*\left(z_{1},z_{2}\right)\right)\\ \;\geq -1-\text{max}\left\{\lambda^{-}\times \sigma^{-}\left(\left(x_{1},x_{2}\right)*\left(\left(y_{1},y_{2}\right)\cdot \left(z_{1},z_{2}\right)\right)\right),\lambda^{-}\times \sigma^{-}\left(y_{1},y_{2}\right)\right\}\\ \;=\text{min}\left\{-1-\lambda^{-}\times \sigma^{-}\left(\left(x_{1},x_{2}\right)*\left(\left(y_{1},y_{2}\right)\cdot \left(z_{1},z_{2}\right)\right)\right),-1-\lambda^{-}\times \sigma^{-}\left(y_{1},y_{2}\right)\right\}\\ \;=\text{min}\left\{{\left(\lambda^{-}\times \sigma^{-}\right)}^{c}\left(\left(x_{1},x_{2}\right)*\left(\left(y_{1},y_{2}\right)\cdot \left(z_{1},z_{2}\right)\right)\right),{\left(\lambda^{-}\times \sigma^{-}\right)}^{c}\left(y_{1},y_{2}\right)\right\}. \end{array}$$

Hence, ♦ ( *λ* × *σ*) is a bipolar fuzzy pseudo-UP ideal of *X* × *Y.*

Conversely, assume that γ( *λ* × *σ*) and ♦ ( *λ* × *σ*) are bipolar fuzzy pseudo-UP ideals of *X* × *Y.* It is clear that *λ* × *σ* is a bipolar fuzzy pseudo-UP ideal of *X* × *Y.* □

## Conclusion

In this paper, we discussed the bipolar fuzzy pseudo-UP ideal of a pseudo-UP algebra and investigated several related properties. We proved that the intersection of two bipolar fuzzy pseudo-UP ideals is bipolar fuzzy pseudo-UP ideal. The union of two bipolar fuzzy pseudo-UP ideals is not always a bipolar fuzzy pseudo-UP ideal. We studied the homomorphic image and the inverse image of the bipolar fuzzy pseudo-UP ideal of pseudo-UP algebra and obtained some interesting results. We proved the Cartesian product of any two bipolar fuzzy pseudo-UP ideals in pseudo-UP algebra and investigated related results. We hope that the findings of this study will add new dimensions to the structures of bipolar fuzzy pseudo-UP ideals based on bipolar fuzzy sets and will serve as a foundation for further study into the structures of fuzzy pseudo-UP ideals by using the concept of bipolar fuzzy subset of pseudo-UP algebra.

## Author contributions

All the authors are contributed equally in this manuscript and also both authors read and approved the final manuscript.

## Ethics and consent

Ethics and consent were not required.

## Data Availability

No data are associated with this article.
