# Supplementary material for: Bipolar Fuzzy Pseudo-UP Ideal Of Pseudo-UP Algebra
Source: F1000Res. 2024 Nov 18;13:1386. [Version 1] doi: 10.12688/f1000research.157173.1 (PMC11809624; doi:10.12688/f1000research.157173.1)
Supplement: Peer review history

# Reviewer report: Reviewer response for version 1

**DOI:** 10.5256/f1000research.172589.r344908

**Published:** 2024-12-26

**Authors:** M Balamurugan

**Affiliations:** 1 Vel Tech, Chennai, Tamil Nadu, India

It is quite interesting to review this article entitled “Bipolar Fuzzy Pseudo-UP Ideal Of Pseudo-UP Algebra”

 In this paper, we apply the concept of bipolar fuzzy sets to pseudo-UP ideals in pseudo-UP algebras. We prove that the intersection of two bipolar fuzzy pseudo-UP ideals is also a bipolar fuzzy pseudo-UP ideal, while the union of two such ideals does not always result in a bipolar fuzzy pseudo-UP ideal. Additionally, we discuss the concepts of bipolar fuzzy pseudo-UP ideals under homomorphism and explore several related properties. The homomorphic image and inverse image of bipolar fuzzy pseudo-UP ideals in a pseudo-UP algebra are also examined in detail. Furthermore, we study the notion of a bipolar fuzzy pseudo-UP ideal under the Cartesian product of pseudo-UP algebra. The Cartesian product of any two bipolar fuzzy pseudo-UP ideals is also the bipolar fuzzy pseudo-UP ideal of pseudo-UP algebra, and then some related results are obtained. However, the paper has been minor revisions in present form but need the following corrections:

1. In page 8, 0.3= ………………(*), remove =
2. In page 9, theorem 3.12 and corollary 3.14, *(* *l* *-* *)* *c*  instead of *(* *l* *-)* *c*  and l = (X, *(* *l* *-* *)* *c* , *l* *-* *)*  instead of  l = (X, *(* *l* *-* *)* *c* ), *l* *-*
3. In page 9, corollary 313,  l = (X, *l* *+* *,*  *(* *l* *+* *)* *c* *)*  instead of l = (X, *l* *+* *,*  *((* *l* *+* *)* *c* *)* . Throughout the paper check it.
4. In page 14, proof of the condition 2) *σ* *-* (y) ≤ *l* *-* (0) instead of *σ* *-* (y) ≤ *l* *-* (y).
5. In references, the recent references to add in the related work. [Ref 1[ and [Ref 2].

Is the work clearly and accurately presented and does it cite the current literature?

Yes

If applicable, is the statistical analysis and its interpretation appropriate?

No

Are all the source data underlying the results available to ensure full reproducibility?

Yes

Is the study design appropriate and is the work technically sound?

Yes

Are the conclusions drawn adequately supported by the results?

Yes

Are sufficient details of methods and analysis provided to allow replication by others?

Yes

Reviewer Expertise:

Fuzzy Algebra

I confirm that I have read this submission and believe that I have an appropriate level of expertise to confirm that it is of an acceptable scientific standard.

## References

[1] : ()-Bipolar Fuzzy-Ideals of BCK/BCI-Algebras. *Journal of Function Spaces* .2021;2021: 10.1155/2021/6615288 1-8 10.1155/2021/6615288

[2] : Bipolar Intuitionistic Fuzzy Soft Ideals of BCK/BCI-Algebras and Its Applications in Decision-Making. *Mathematics* .2023;11(21) : 10.3390/math11214471 10.3390/math11214471

# Reviewer report: Reviewer response for version 1

**DOI:** 10.5256/f1000research.172589.r344905

**Published:** 2024-12-24

**Authors:** Ubaid Ur Rehman

**Affiliations:** 1 University of Management and Technology,, Lahore, Punjab, Pakistan

I have read the manuscript thoroughly and decided that the idea is new but the paper in recent shape cannot be accepted. I am adding some specific comments below. So, my decision regarding the current version of the manuscript is a major revision.

 Comments:

1. The abstract should be improved. The present abstract is not up to the mark.  
2. The motivation of the proposed theory need a lot of improvement. The authors must discuss that what is the requirement of this theory to be developed.
3. The authors interpreted the concept of bipolar valued algebraic structure, so they must discuss some recent work on fuzzy groups such as Bipolar Complex Fuzzy Subgroups [ref 1], Bipolar Complex Fuzzy Semigroups [ref 2], Analysis of Γ-Semigroups Based on Bipolar Complex Fuzzy Sets [ref 3] , T-Bipolar Soft Groups and Their Fundamental Laws [ref 4], Bipolar complex fuzzy submodules [ref 5] in the introduction.  
4. There must be suitable punctuation mark after every equation.
5. The theoretical work is okay but what is the application of the proposed work? The authors must add an application of the bipolar valued fuzzy groups to show the practicality of their work.
6. Improve the language of the whole manuscript with the help of native English speakers, and the structure of each section to ensure the readability of the manuscript. 
7. The conclusion is written like a contribution, but there is no deep analysis of obtained knowledge, no discussion, or wider application.
8. How the authors can expand their work to other mathematical frameworks such bipolar complex fuzzy set devised by Tahir Mahmood? The author should discuss it in the future direction by citing 2 to 3 articles of bipolar complex fuzzy set for better future work.
9.  Add some merit and limitations of the proposed work.

Is the work clearly and accurately presented and does it cite the current literature?

No

If applicable, is the statistical analysis and its interpretation appropriate?

I cannot comment. A qualified statistician is required.

Are all the source data underlying the results available to ensure full reproducibility?

No source data required

Is the study design appropriate and is the work technically sound?

Partly

Are the conclusions drawn adequately supported by the results?

Partly

Are sufficient details of methods and analysis provided to allow replication by others?

Partly

Reviewer Expertise:

fuzzy algebra, decision making, algebraic structures

I confirm that I have read this submission and believe that I have an appropriate level of expertise to confirm that it is of an acceptable scientific standard, however I have significant reservations, as outlined above.

## References

[1] : Bipolar Complex Fuzzy Subgroups. *Mathematics* .2022;10(16) : 10.3390/math10162882 10.3390/math10162882

[2] : Bipolar complex fuzzy semigroups. *AIMS Mathematics* .2023;8(2) : 10.3934/math.2023200 3997-4021 10.3934/math.2023200

[3] : Analysis of $$Gamma $$-semigroups based on bipolar complex fuzzy sets. *Computational and Applied Mathematics* .2023;42(6) : 10.1007/s40314-023-02376-w 10.1007/s40314-023-02376-w

[4] : T-Bipolar soft groups and their fundamental laws. *Journal of Intelligent & Fuzzy Systems* .2024;46(4) : 10.3233/JIFS-236150 9479-9490 10.3233/JIFS-236150

[5] : Bipolar complex fuzzy submodules. *Physica Scripta* .2024;99(6) : 10.1088/1402-4896/ad421a 10.1088/1402-4896/ad421a

# Reviewer report: Reviewer response for version 1

**DOI:** 10.5256/f1000research.172589.r344899

**Published:** 2024-12-18

**Authors:** Baravan Asaad

**Affiliations:** 1 Cihan University-Duhok, Duhok, Iraq; 2 University of Zakho, Zakho, Kurdistan, Iraq

The manuscript presented a new concept of pseudo-UP algebras called bipolar fuzzy pseudo-UP ideal which is combine between bipolar fuzzy sets and pseudo-UP ideal. Some theoretical results are presented via this new concept of pseudo-UP algebras.

 I have only the following points:

 - What is the comparison between the existing concept and the previous concept?

 Therefore, I recommend to accept this manuscript for indexing.

Is the work clearly and accurately presented and does it cite the current literature?

Yes

If applicable, is the statistical analysis and its interpretation appropriate?

Not applicable

Are all the source data underlying the results available to ensure full reproducibility?

Yes

Is the study design appropriate and is the work technically sound?

Yes

Are the conclusions drawn adequately supported by the results?

Yes

Are sufficient details of methods and analysis provided to allow replication by others?

Partly

Reviewer Expertise:

Topology, Fuzzy Set Theory, Soft Set Theory, Decision Making.

I confirm that I have read this submission and believe that I have an appropriate level of expertise to confirm that it is of an acceptable scientific standard.
